# Dynamic Landscape Analysis of cell fate decisions provides predictive models of neural development from single-cell data

**DOI:** 10.1371/journal.pbio.3003953

**Published:** 2026-08-26

**Authors:** Marine Fontaine, M. Joaquina Delás, Meritxell Sáez, Rory J. Maizels, Elizabeth Finnie, James Briscoe, David A. Rand

**Affiliations:** 1 Mathematics Institute, University of Warwick, Coventry, United Kingdom; 2 Zeeman Institute for Systems Biology and Infectious Epidemiology Research, University of Warwick, Coventry, United Kingdom; 3 The Francis Crick Institute, London, United Kingdom; 4 IQS, Universitat Ramon Llull, Barcelona, Spain; California Institute of Technology, UNITED STATES OF AMERICA

## Abstract

Building a mechanistic understanding of cell fate decisions remains a fundamental goal of developmental biology, with implications for stem cell therapies, regenerative medicine and understanding disease mechanisms. Single-cell transcriptomics provides a detailed picture of the cellular states observed during these decisions, but building dynamic and predictive models from these data remains a challenge. Here, we present *dynamic landscape analysis* (DLA), an integrative framework that applies dynamical systems theory to identify stable cell states, map transition pathways, and generate a predictive cell fate decision landscape from single-cell data. Applying this framework to vertebrate neural tube development revealed that progenitor specification by Sonic Hedgehog (Shh) can be captured in a landscape with an unexpected topology in which initially divergent lineages converge to the same fate through multiple distinct routes. The model accurately predicted cellular responses and cell fate allocation for unseen dynamic signalling regimes. Cross-species validation using human embryonic organoid data demonstrated conservation of this decision-making architecture. By modelling the dynamic responses that drive cell fate decisions, the DLA framework provides a quantitative and generative framework for extracting mechanistic insights from high-dimensional single-cell data.

## Abstract 2

Applying dynamical systems theory to single-cell data can provide a detailed picture of the cellular states observed during cell fate decisions. This study presents a dynamic landscape analysis (DLA) as an integrative framework that reveals how morphogen signals orchestrate robust cell fate decisions during neural tube development across species.

## Introduction

During embryonic development and tissue homeostasis, cells make sequential fate decisions to produce the diverse sets of specialised cell types that form functional tissues. These decisions are driven by gene regulatory networks (GRNs) that interpret external signals and coordinate cell-type-specific gene expression programmes [1]. Understanding how signalling inputs and GRNs coordinate these fate decisions remains a fundamental challenge in biology.

Recent work has approached this problem by using dynamical systems theory to formalise Waddington’s pioneering metaphor [2] of cellular differentiation as balls rolling down a landscape of branching valleys [3–25]. This view rests on a correspondence between cell state transitions and rigorously defined entities in dynamical systems (Box 1). Cell fates correspond to *attractors*, the stable states of the dynamical system generated by the GRN. Transitions between states occur through signal-driven bifurcations that destabilise the current state [3,5,10,26–28] and lead cells to follow a mathematically defined path of steepest descent, analogous to a valley bottom in Waddington’s landscape, as they descend towards a new attractor [17,19].

Despite these advances, relatively few systems have been analysed this way, so it remains unclear whether the theoretically predicted bifurcations, which fall into a limited number of generic types [17,29], are sufficient to describe cell fate decisions. In part, this is because constructing quantitative landscape models from high-dimensional scRNA-seq data is hard: identifying dynamical features such as attractors and unstable manifolds requires working in gene-expression space, yet current practice [30,31] typically selects all highly variable genes before reducing dimension with linear and nonlinear projections such as principal component analysis (PCA) and UMAP. This presents critical obstacles for the dynamical systems analysis of cell fate transitions. The set of highly variable genes is sensitive to the choice of normalisation [32,33], and most of these genes lie outside the small subset that governs GRN dynamics [1]. The resulting gene-expression space is therefore poorly defined, it depends on preprocessing choices and is dominated by features irrelevant to the decision-making process.

Here, we present *dynamic landscape analysis* (DLA) a framework that addresses these challenges by providing tools to identify the structure of a dynamical landscape and to construct quantitative models from high-dimensional single-cell data. Our approach finds cell states and transition pathways by identifying gene expression spaces of reduced dimension d≈ 50–100 that preserve the structural details of higher-dimensional scRNA-seq data while explicitly including the genes governing transition dynamics. We avoid the use of nonlinear projections or ones that do not preserve the smoothness of trajectories and use simple linear 3D visualisations that maintain direct connections to individual gene expression levels, enabling computationally tractable and biologically interpretable analysis.

We applied DLA to an in vitro system of ventral neural tube development. In this tissue, progenitor cells organise into discrete gene expression domains linearly arrayed along the dorsal-ventral axis. The positioning of these domains is determined by Sonic Hedgehog (Shh) signalling from the ventral pole. This developmental patterning can be recapitulated in vitro using mouse embryonic stem cells exposed to different levels and duration of Shh signalling [34–36] (Fig 2A). Profiling transcriptomes of individual cells during this differentiation process offers a high-resolution view of cell states and fate transitions.

Our analysis revealed both well-characterised progenitor states and previously unappreciated intermediates, situated within a complex landscape controlled by Shh signalling. We discovered an unexpected topology involving connections between multiple cell states where initially divergent lineages converge to the same set of fates through distinct routes. We used these insights to construct a mathematical model that accurately reproduced the observed cell fate decisions.

The model revealed several key features of neural tube patterning that suggest general principles. The model identified the bifurcation mechanisms underlying branching decisions, confirming that simple, theoretically predicted bifurcations are ubiquitous. Moreover, model-guided experiments showed how Shh signalling level and timing control both the proportions of cell types and the irreversibility of the bifurcations that destabilise cell states.

The findings challenge the conventional view of morphogen tissue patterning, in which boundaries between distinct domains form through monotonic morphogen concentration thresholds (the French flag mechanism [37]), implying that there is a direct differentiation pathway connecting the cell states on either side of the boundaries. Our results suggest that two spatial boundaries between progenitor gene expression domains form because of branching decisions, represented by flip bifurcations, with no such direct differentiation pathway connecting the cell state. This reframes our understanding of how morphogen gradients establish patterning thresholds. Rather than operating purely through simple concentration-dependent switches, Shh signalling organises spatial pattern hierarchically, thereby expanding the repertoire of developmental patterning mechanisms.

Finally, analysis of human embryonic organoid scRNA-seq data [38] demonstrates that this decision-making architecture is conserved between mouse and human. The dynamical landscape model accurately predicted cell state proportions despite species-specific differences in developmental timing and identified which decisions in the network were affected by notochord-derived Shh. This cross-species validation establishes the fundamental nature of the identified landscape topology in ventral neural progenitor specification.

**Box 1. A dynamical-systems view of cellular decision-making: attractors and saddles. Terms explained: attractors, attractor clusters, saddles, unstable manifold, transition route, near-bifurcation escape, fold bifurcation, basins of attraction, microheterogeneity, landscape.**

We interpret cell fate decisions using dynamical systems theory applied to gene regulatory networks (GRNs). Molecular interactions define a stochastic dynamical system governing cell state evolution, while external signals reshape the available states and transitions by modulating system parameters. The central structures are *attractors*, *saddles*, and the *transition routes* connecting them, together forming a *dynamical landscape*.

The GRNs considered here are strongly dissipative, so trajectories converge to stable fixed points corresponding to cell fates; we do not consider oscillatory states. Although gene expression is noisy, the noise is usually insufficient to destroy the underlying deterministic structure. We therefore treat cellular dynamics as a stochastic perturbation of a deterministic system: the deterministic component defines the possible states and connections, while stochasticity influences the timing and route of transitions.

**Fig 1:**
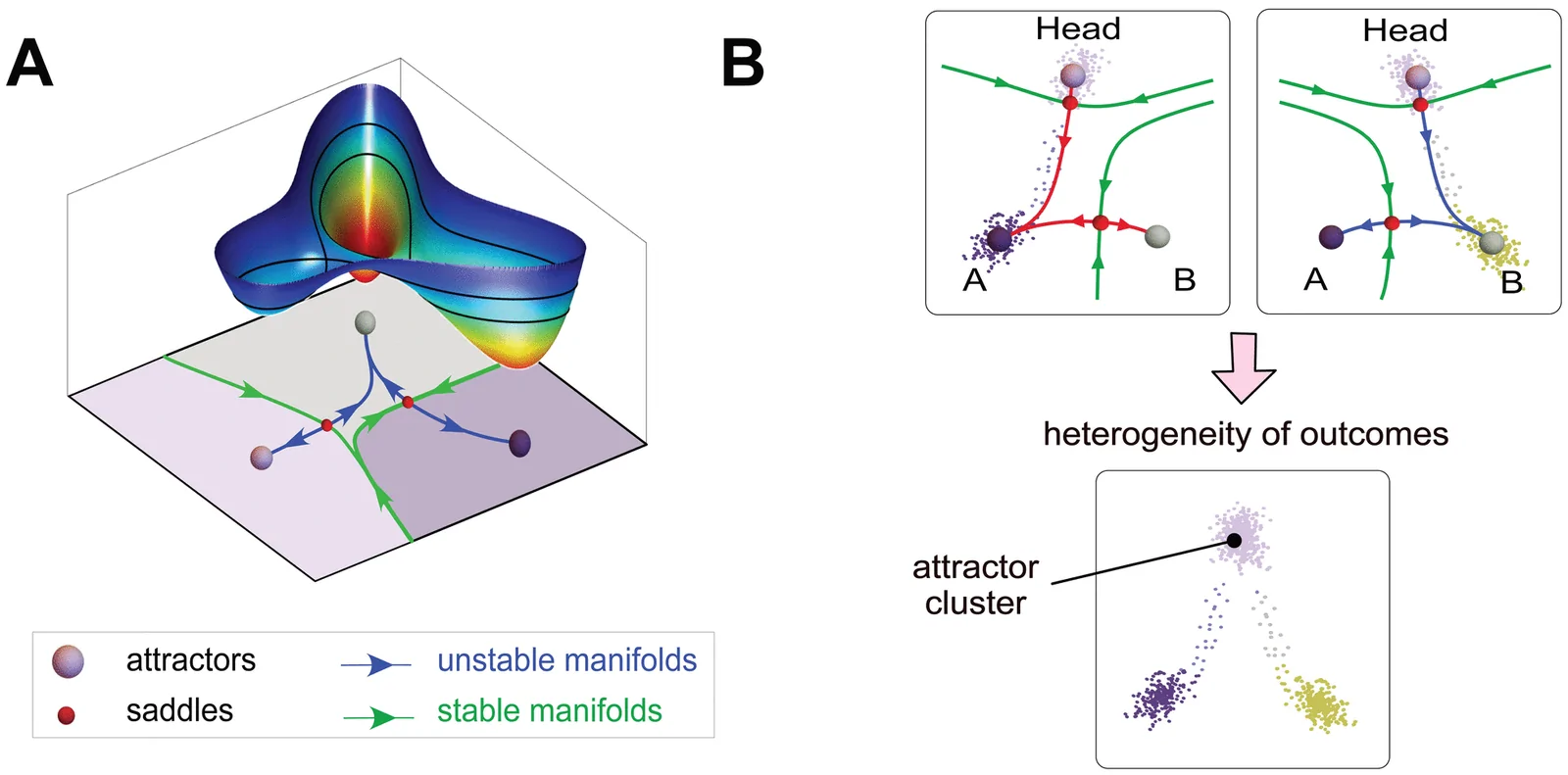
Dynamical systems terminology. A. A three attractor system. Blue curves are the unstable manifolds of the saddles (red balls) and the green curves are stable manifolds. **B.** When stochastic, attractors become clusters and stable manifolds are the splines of the transitioning routes. Heterogeneity of the cells in the head attractor H implies that their dynamics is driven by distinct landscapes, one with unstable manifold connecting to A and other connecting to B.

**Attractors and cell fates.** Stable cell fates correspond to deterministic *attractors* (Fig 1A). Noise causes cells to fluctuate around them, producing clouds of gene-expression profiles we term *attractor clusters* (ACs) (Fig 1B).

We identify ACs through properties expected of attractors: incoming transition routes, persistence across time points, approximately unimodal expression distributions, loss of stability via characteristic bifurcations, and transitions to neighbouring ACs.

**Saddles and transition routes.** Certain saddles act as “mountain passes” enabling transitions between fates. We focus on saddles with a one-dimensional unstable manifold, which act as passes in the landscape. Transitions occur either through *near-bifurcation escape*, where stochastic fluctuations drive escape, or when a signal change causes the saddle and attractor to collide in a *fold bifurcation*, destroying the attractor. In both cases the most likely route over the saddle follows the saddle’s *unstable manifold*—the curve of points whose infinite-backward-time limit is the saddle. Such a well-defined route also exists when the attractor is destroyed in a fold.

Because the system is stochastic, cells only approximately follow this unstable manifold and in data this single path is replaced by a *transition route* that approximates it.

The proximity of an attractor and its neighbouring saddle determines the attractor’s *depth*. Cells in deep attractors require large perturbations to escape and therefore represent robust fates, whereas cells in shallow attractors are easily destabilised. Signals alter depth by changing GRN parameters, allowing stable fates to become unstable.

**Stable manifolds and basins of attraction.** The complementary *stable manifolds* (green curves in Fig 1A) partition the phase space into *basins of attraction*, regions whose trajectories converge to the same attractor (coloured regions in Fig 1A). Crossing a stable manifold corresponds to a commitment event.

Because cells in a given cell state have a slightly different molecular state, cell populations contain microheterogeneous landscapes. Apparently similar cells may therefore adopt different fates not only through stochastic fluctuations but also because they occupy different sides of basin boundaries or bifurcation curves (Box 3). The extra stochasticity introduced by microheterogeneity further smears the ACs and transition routes.

**Head attractor clusters.** At branching decisions there is always an upstream AC from which cells can access multiple downstream fates; we term this the *head AC*. Microheterogeneity and signalling differences within the head AC generate distinct escape trajectories, producing branching behaviour (Fig 1B).

**Landscapes and topology.** The attractors, saddles, and connecting unstable manifolds together define the dynamical landscape (Fig 1A). Its graph structure—with attractors as nodes and transition routes as edges—can possess nontrivial topology.

## Results

### Identification and validation of attractor clusters in scRNA-seq data

**Fig 2:**
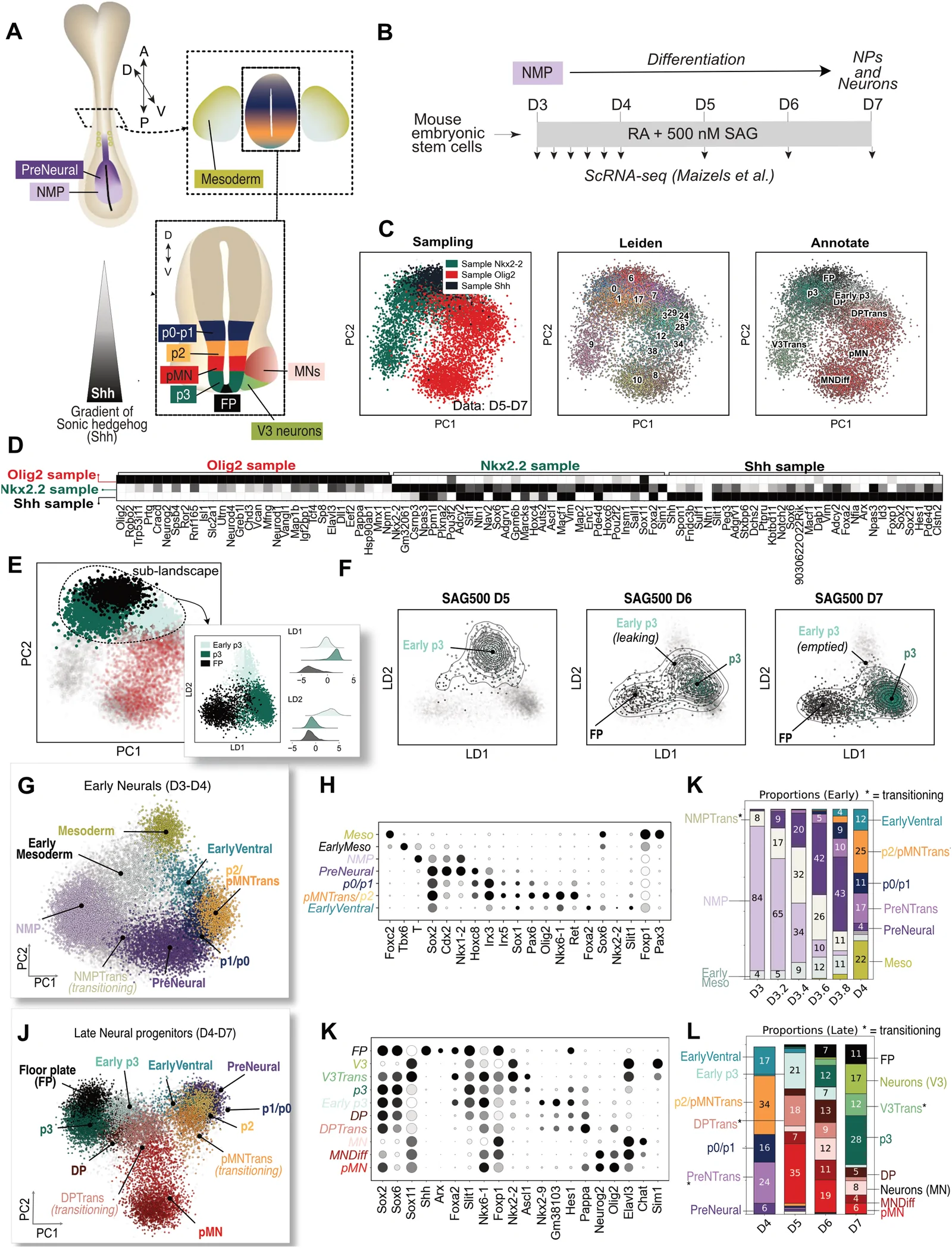
Attractor clusters in single-cell RNA sequencing data reveal discrete cell states during neural development. **A.** Schematic diagram of neural tube patterning regulated by the Shh gradient. **B.** Schematic of in vitro differentiation of mESCs and timing of the scRNA-seq data collection (D, Day). **C.** Example of the outward clustering methodology illustrated by analysis of the late time points (D5-D7). (Left) 2D PCA projection of cells that express one of the marker genes Olig2, Nkx2.2 and Shh. (Middle) PCA projection from *d*-dimensional effective gene space using the *d* most significantly differentially expressed genes between the 3 samples of cells (here *d* = 112). Cells are clustered using Leiden clustering (a graph-based algorithm for identifying densely connected groups of cells) with fine resolution and coloured accordingly. (Right) The fine Leiden clusters are merged based on their molecular identities. **D.** The most significantly differentially expressed genes distinguishing the 3 samples of cells shown in C (left panel). **E.** Extraction of the group of adjacent ACs, Early p3, p3 and FP, visualised in 2D LDA space. Histograms for each linear discriminant score (i.e., the dot product of the data vector with the ith LDA eigenvector) show that ACs (such as FP) exhibit a well-defined approximately Gaussian structure in LDA space. **F.** Temporal progression of Early p3, p3, and FP identifies Early p3 as the ("head") state from which cells transition to either p3 or FP. Grey dots represent all cells in the group (D5-D7), coloured dots indicate cells present at the indicated time points with the colour indicating their cluster assignment. Contour lines indicate the density of cells at each time point. **G–H.** Identification of early ACs (D3-D4) and transitioning cells between them labelled -Trans. G. PCA projection of cells from D3-D4 samples showing the early ACs. H. Dot plot indicating the expression of markers used to identify the early ACs. The size of the circle represents the percentage of cells in the AC and the intensity of shading indicates the mean expression of the gene. **I.** The proportion of cells (in percent) in each AC and transitioning clusters at the early time points (D3-D4). **J–L.** Identification of late ACs (D4-D7) and transitioning cells between them, labelled as -Trans. Similar to G**–**I but using cells from the D4-D7 samples. The D8 sample is not displayed; it predominantly contains MNs, V3 neurones, MNDiff cells, and p3 cells. The data underlying this Figure can be found at https://doi.org/10.5281/zenodo.15584010.

**Fig 3:**
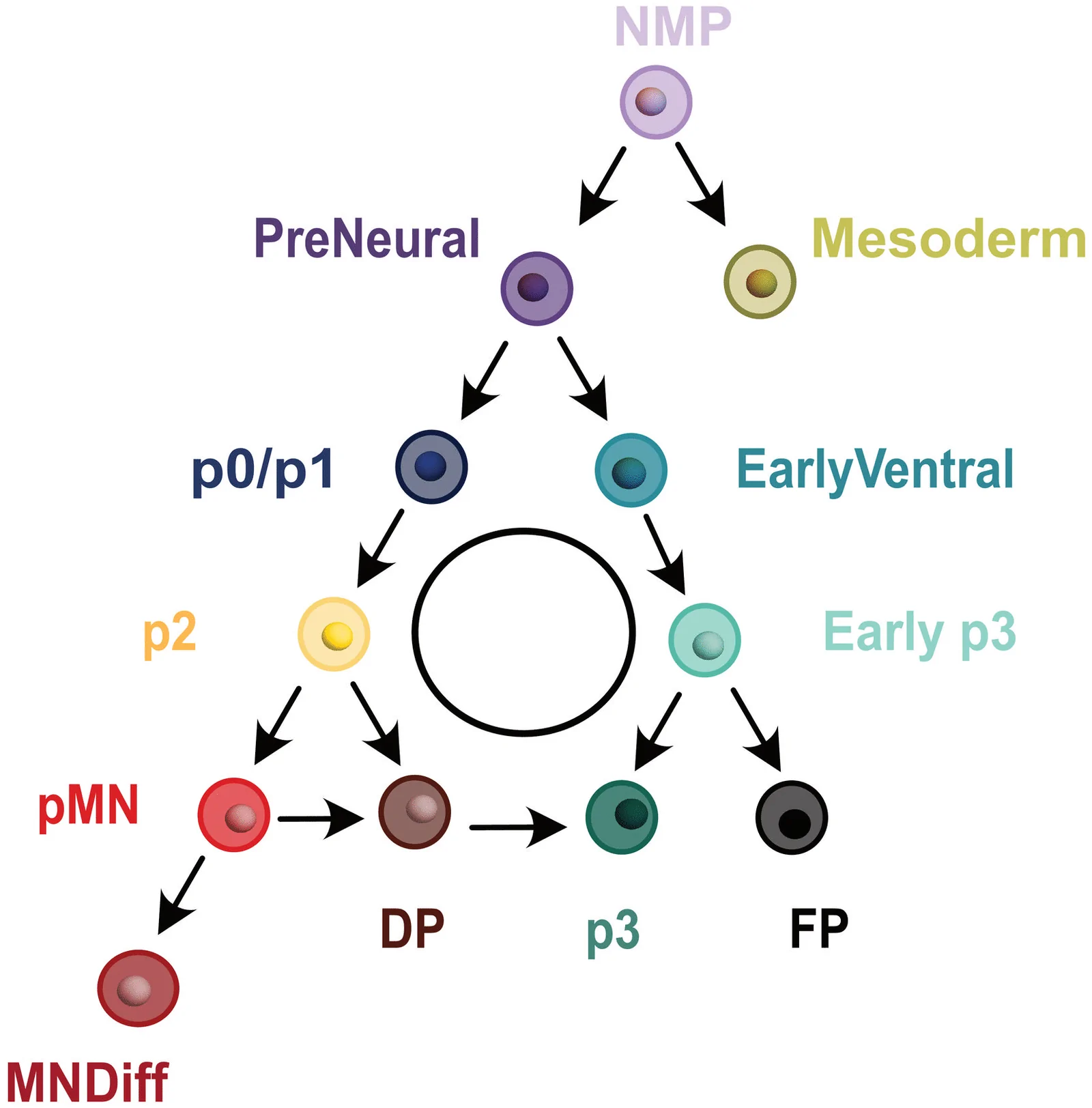
Neural tube decision landscape. Schematic diagram summarising all the ACs associated with the cell states found in the scRNA-seq dataset at 500 nM SAG across all time points (D3–D7). This is included to introduce the complete set of states in the landscape and the possible transitions between them. These will be discussed in later sections.

To prototype our approach, we took advantage of an in vitro system in which pluripotent mouse embryonic stem cells were differentiated into distinct neural progenitor subtypes in response to Sonic Hedgehog signalling [36,39,40] (Fig 2A). We analysed a scRNA-seq dataset [40] comprising approximately 40,000 cells across 10 time points, generated in response to continuous exposure to 500 nM SAG, a Shh signalling agonist (Fig 2B). Based on prior knowledge of ventral neural tube development [36,39–42], we expected to identify neuromesodermal progenitors (NMPs) [34], the PreNeural state [43] linking NMPs to neural fates, mesoderm cells [34], and the ventral neural tube progenitor subtypes pMN, p3, and floor plate (FP) [42] (Fig 3). We anticipated that p0/p1 and p2 neural progenitor states might be present but rare at this SAG concentration [36,40,44].

#### Outward clustering identifies cell states through iterative gene selection.

We developed an iterative gene selection and clustering approach that we term *outward clustering*: beginning from a small set of known marker genes, the method iteratively expands—or “clusters outward”—by identifying genes that are differentially expressed between the clusters defined at each step, progressively building up the effective gene space without presupposing any cell states in advance. The approach is based on the principle that genes relevant to the GRN should show differential expression between cell states or transition pathways (detailed in Section A1.1 in S1 Appendix). Since cell states are regarded as initially unknown, we bootstrapped the analysis using a small number of marker genes (typically three or four) that define known cell types, regions or pathways. For early time points (D3-D4), we used TBXT/BRA, Foxc2, Foxa2, and Olig2, which identify neuromesodermal progenitors, mesodermal tissues, and early neural lineages [36]. We identified subsets of cells expressing just one of these markers and performed differential gene expression analysis between these subsets. This process generated gene modules of approximately 100 genes, determined by setting statistical significance thresholds. For later time points, we used Olig2, Nkx2.2, and Shh which are markers of pMN, p3, and FP progenitors and performed an equivalent analysis (Fig 2C, 2D).

Although the early and late gene modules produced in this way define relatively high-dimensional data spaces (which we call *effective gene spaces*), they are reduced in dimension by a factor of about 30 compared to the dimensionality of all highly variable genes. This allowed almost all analyses to be carried out in gene space, as advocated in [45], without resorting to low-dimensional projections, except for visualisation. This, for example, allowed us to compute cell fate differentiation paths in the effective gene spaces as opposed to tracing them on a 2D or 3D visualisation. The only exception was for the fine-resolution Leiden clustering for which we projected the data into a lower-dimensional PCA space [46]. Following this, we merged fine-scale clusters into cell state clusters based on the molecular identities that were identified (Fig 2C).

We confirmed that we had captured the genes that are differentially expressed between each cluster and the others. We checked for missed clusters by examining unassigned cells for potentially missed states and testing whether additional genes showed significant differential expression between cluster pairs. We also confirmed robustness by substituting alternative correlated marker genes and verifying that cluster structures remained consistent. After analysing transition pathways (described below), we expanded the gene set to include genes showing significant variation in transitions, along with expert-curated transcription factors. None of these additions substantially affected subsequent analyses.

#### Validation of attractor clusters.

In a deterministic system, a stable gene expression state (i.e., an attractor) would be a single fixed point: a precise combination of gene activity levels maintained by regulatory feedback. The stochastic nature of molecular dynamics and the data modality causes cells states to fluctuate around this point, replacing it with a cloud of similar expression profiles. We call this cloud an *attractor cluster* (AC). It has key properties that can be used to validate its status: it captures nearby states, has a well-defined unimodal statistical structure, will either be stable or bifurcate in characteristic ways, and will connect via saddle points to one or more upstream and downstream ACs.

For this validation we used multiple criteria. Firstly, we tracked each AC over time to assess its stability at successive time points, identify any loss of stability, and characterise resulting transitions to adjacent ACs. Secondly, in order to clarify the topological relationship between ACs and more clearly see the relationship between an AC and neighbouring ACs connected by transition routes, we extracted small groups of neighbouring ACs and their associated transitioning clusters – which we term *sub-landscapes*– and projected them into 2- or 3-dimensions using linear discriminant analysis (LDA) [47]. LDA maximises separation between clusters and faithfully represents them because, although LDA rescales axes, it is linear and therefore does not introduce artificial bends, folds, or discontinuities and faithfully maintains the topological relationships beetween states (and how routes connect them). To illustrate this approach, consider the previously unappreciated cell state represented by the Early p3 AC together with the p3 and FP ACs (Fig 2E). These together with the two transition routes from Early p3 make up such a sub-landscape where the Early p3 AC loses stability causing cells to transition to either p3 or FP, consistent with published observations [48] (Fig 2F).

Thirdly, Wilcoxon rank-sum tests confirmed that known progenitor marker genes distinguished ACs. Fourthly, we measured Kullback-Leibler divergence (a statistical measure of how well-separated two clusters are) in LDA space (Table A1 in S1 Appendix), which provides a lower bound for the same divergence measured in the effective gene space. Fifthly, cell distributions within each AC were checked to be unimodal along linear discriminant components, consistent with approximately linear dynamics near attractors (Fig 2E). Finally, we examined gene-gene correlations within and between ACs, with differing correlations providing evidence of distinct regulatory interactions in each cluster (Section A1.3 in S1 Appendix).

An important aspect of our analysis is the reproducibility of our determination of ACs. We have data across multiple timepoints and, for the flow cytometry data, which is discussed below, across multiple experimental conditions. Directly comparing the different ACs across these conditions indicated that they are reproducible, as can be appreciated by comparing data at a given time point and/or experimental condition with the position of the dataset consisting of all times and conditions. To visualise this when projecting into a lower dimensional space we always show the data of current interest against a grey plot of all the data in that sub-landscape. This reproducibility provides additional evidence to determine the persistence of ACs and the location of transition routes. These reproducibility checks are performed in gene expression space, LDA projections and on the standard 2D plots of expression levels of pairs of key genes. As an example consider Fig 5A, 5B below, showing the branching decision from NMP. This shows the overall structure, the emptying of the NMP state and filling of the Early Mesoderm and PreNeural ACs and the stability of their position over four time points.

This method identified all expected cell states plus several previously uncharacterised intermediates which span early mesoderm specification to ventral neural progenitors [34] (Figs 2G – 2L and 3).

At early time points, ACs included NMPs [49] (TBXT/Bra), mesodermal cells [50] (Foxc2), PreNeural cells [43,51] (Nkx1.2, Msx1, Sox2), transitioning NMPs (NMPTrans), and Early Mesoderm [52,53] (Tbx6). As differentiation progressed, ventral neural progenitor states emerged: pMN [54] (Olig2), p3 [55] (Nkx2.2), and FP [56] (Foxa2, Arx, Shh). The p0/p1 (Pax6, Irx3) and p2 (Pax6, Irx3, Nkx6.1) ACs appeared at low numbers at D3.8 and D4, consistent with high SAG concentrations.

Additional ACs/cells states and genes that characterise them included MNDiff (Olig2, Neurog2), representing pMN progenitors undergoing motor neurone differentiation [44,57], and DP (Olig2, Nkx2.9, Hes1), representing an intermediate between p3 and pMN identities [58]. The EarlyVentral population (Nkx6.1, Foxa2) at D3.8-D4 represents a common precursor of FP and p3 [36]. At D5, we identified Early p3 (Nkx2.9), distinct from conventional p3 (Nkx2.2, Ascl1) observed at D6-D7 [48,59,60], and V3 neurones (Sim1, Nkx2.2), the differentiated p3 progeny [55].

Overall, this analysis demonstrates that outward clustering robustly identifies all known cell states and reveals developmental intermediates.

### Analysis of cell state transitions using unstable manifold mapping

We next set out to validate transition pathways and the bifurcations underlying branching trajectories. These bifurcations and their relation to decision-making are described in Box 2. Cells escape their current AC either because the AC is annihilated in a fold bifurcation or because stochastic fluctuation causes a near-bifurcation escape (Box 1). Where branching decisions are indicated, we determine the nature of the bifurcations regulating the branching. The two simplest such mechanisms are discussed in Box 2.

**Box 2. A dynamical-systems view of cellular decision-making: bifurcations. Terms explained: fold bifurcation, flip bifurcation = heteroclinic bifurcation, binary flip decision, binary choice decision, genericity.**

A central mechanism by which signals induce fate changes is through *bifurcations*, where the structure of the landscape changes qualitatively. Cell fate decisions arise because signals move the system through parameter space, reshaping the landscape: an attractor may disappear removing a stable cell fate or forcing a transition. Alternatively, a transition route may change, leading to a different cell fate.

**Fig 4:**
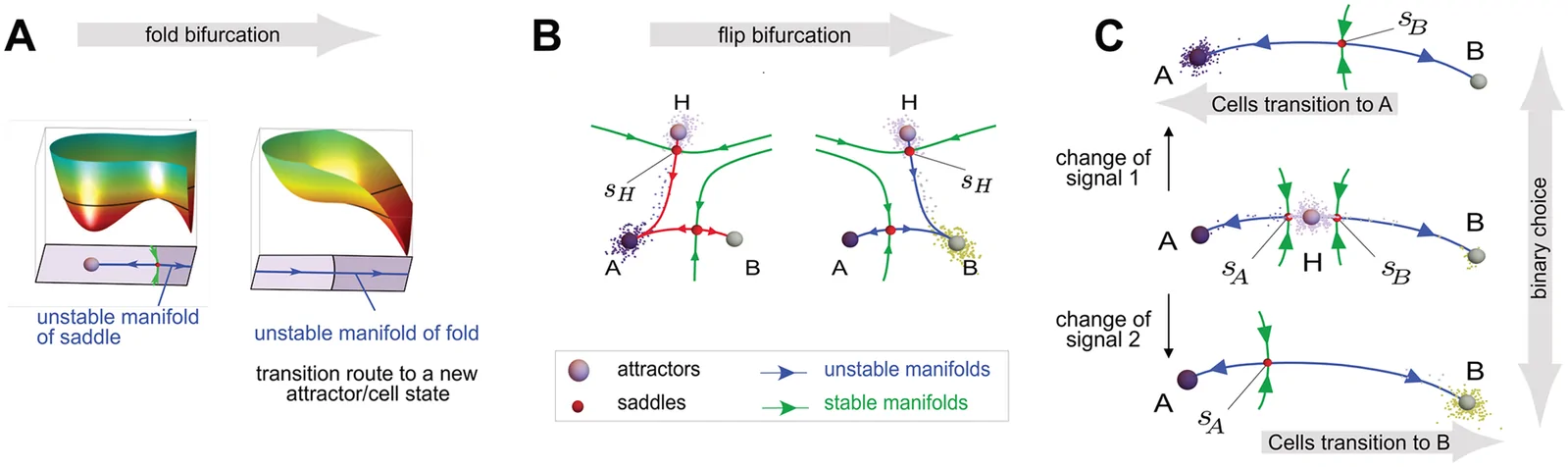
Dynamical-systems view of decision making. A. The fold bifurcation. (Left) before the bifurcation an attractor and a saddle are close. (Right) at the bifurcation, a well-defined unstable manifold still determines the transition route the cells will follow. **B.** The flip bifurcation. The unstable manifold of the saddle flips from connecting the head attractor *H* to *A* so that it connects *H* to *B*. The change might be caused by a signal change or effect of neighbouring cells on the cell. **C.** The binary choice landscape. A central attractor is separated from two adjacent attractors by independent saddles. A change in signal produces a fold bifurcation of the central attractor by one or other saddle, resulting in cells making a direct transition from the central attractor along the unstable manifold to the peripheral attractor.

**Bifurcations that create or destroy stable states: folds.** (Fig 4A) The simplest and most important example is the *fold bifurcation* (also called a *saddle-node*), in which an attractor and a saddle collide and annihilate each other. In developmental terms, a previously stable cell state then disappears, and the cells that occupied it are forced towards an alternative fate. We refer to such events as *bifurcational cell state transitions*.

**Bifurcations that change the transition pathways: flips.** (Fig 4B) A *flip bifurcation* (also called *heteroclinic bifurcation*) occurs when the unstable manifold (the escape trajectory) of the saddle near the head attractor *H* switches its connection from one downstream attractor to another, typically a bistable pair. A binary flip decision is built on this structure: three attractors and two saddles, with cells held at *H* until bifurcation or stochastic fluctuations drive them towards one of the two downstream attractors, the choice depending on signals and cell state. Because the escape is governed by a single saddle, cells exit along a nearly shared initial direction before diverging, so transitioning cells at the earliest exit stage form a single cluster rather than two (the latter being the signature of a binary choice decision).

**Bifurcations choosing between one of two obligatory fates: binary choice landscape.** (Fig 4C) Here, three attractors and two saddles sit in a row (Fig 4C). Either of the two saddles on either side of the central attractor can annihilate it, and cells in this precursor state then escape to the attractor connected to the saddle involved [18].

**Why these mechanisms and structures are universal.** The key idea is *genericity*: a mathematically rigorous concept capturing that, within broad classes of systems, only a limited set of behaviours are robust, while others require fine-tuning. For GRN-like systems, this strongly constrains the possible landscape structures. In particular, under variation of a single parameter, and except in exceptional circumstances, attractors are lost through fold bifurcations and transition routes are changed through flip bifurcations.

#### Direct transitions and binary decisions.

**Fig 5:**
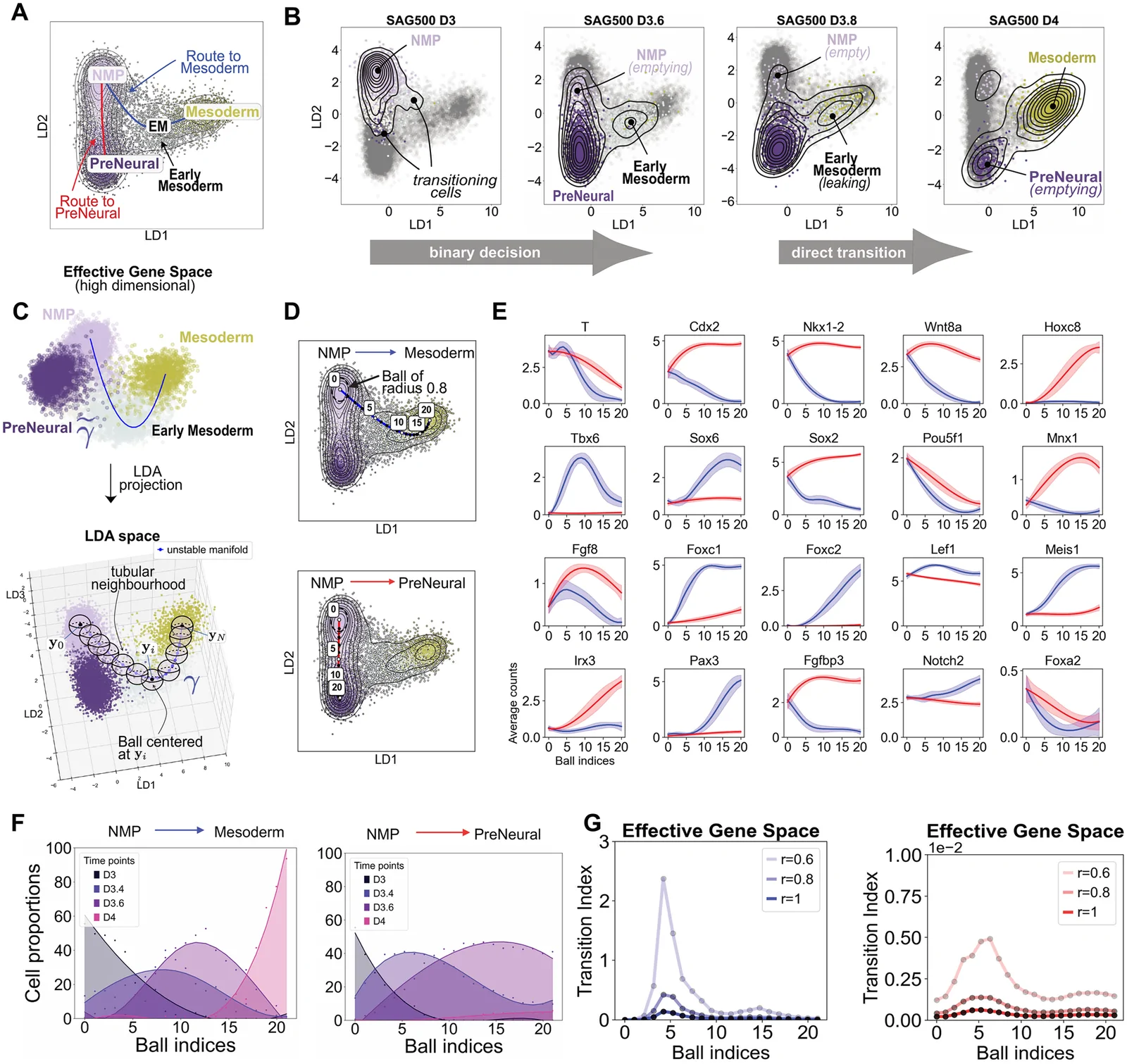
Analysis of cell state transitions using unstable manifolds between attractor clusters. **A.** Binary decision from NMP to PreNeural and Early Mesoderm/Mesoderm. The two approximated unstable manifolds (red and blue curves) for the landscapes of cells projected into LDA space. The red approximated unstable manifold outlines the route to PreNeural and the blue unstable manifold the route to Early Mesoderm and Mesoderm. **B.** Temporal progression of the NMP, PreNeural, Early Mesoderm and Mesoderm ACs in LDA coordinates. Grey dots represent all cells in the group, coloured dots indicate cells present at the indicated time points with the colours indicating their cluster assignment. Contour lines indicate the density of cells at each time point. This exemplifies the binary flip decision from NMP to PreNeural or to Early Mesoderm, followed by a direct transition from Early Mesoderm to Mesoderm. At D3 almost all cells (dots) are in the NMP AC. Cells in both PreNeural and Early Mesoderm (Tbx6+) are evident at D3.6, and increase at D3.8. There is then a direct transition from Early Mesoderm to Mesoderm at D3.8 and PreNeural empties as cells transition to other states (see Fig A1 G in S1 Appendix). **C.** (Top) The unstable manifolds are estimated in the higher dimensional effective gene space. This avoids artificial ordering of the points along the curve that might occur if, for example, the LDA components were strongly determined by a single gene. This curve is then projected into the LDA space of interest (2D or 3D). (Bottom) Balls are placed along the projected curve in LDA space. Together they form a tubular neighbourhood of the unstable manifold. Raw counts (or, equivalently, log-normalised counts) of individual genes are averaged over the cells inside each ball to determine how gene expression changes along the unstable manifold. This construction ensures that the change in expression levels does not depend upon the particular LDA projection used. **D.** LDA plots of the binary flip decision from NMP to either PreNeural or Early Mesoderm using D3-D4 data showing the projected unstable manifolds. The positions of the balls are shown by the index of their centres. A ball of radius 0.8 centreed at index 0 is shown (dashed black). **E.** The variation of key genes along the estimated unstable manifolds from D. Genes such as Cdx2, Sox2, Nkx1.2 and Tbx6 show diverging behaviour along the two routes consistent with the expected behaviour in a binary flip landscape. The gene tendencies are found using 20 random cell samplings of 12% of the cells. **F.** Temporal progression of cells along the projected unstable manifolds in D. Proportions of cells (dots) at stages D3, D3.4, D3.6, and D4 within each ball (of radius 0.8) along the unstable manifolds connecting NMP to Mesoderm (left) and NMP to PreNeural (right). We used a high smoothing factor to obtain smooth densities. In the right panel, there are very few cells at D4 (pink density) because cells at this stage have exited the PreNeural AC (see Fig A1 G in S1 Appendix). **G.** Critical Transition index [61] computed in each ball along the approximated unstable manifolds show peaks as cells transition from one AC to another. Colour variations indicate different ball radii. The data underlying this Figure can be found at https://doi.org/10.5281/zenodo.15584010.

When a near-bifurcation escape takes place, the simplest outcome is that all cells leaving an attractor follow the same path to the same downstream attractor. These *direct transitions* involve no branching and commit cells to a single fate. The next case is a choice between two potential transition routes, whose minimal architecture is the *binary flip landscape* defined in Box 2. In such a landscape, when cells escape the head attractor the choice between fates depends on the signals received and the cell’s state, and changes in either can redirect the route to the alternative downstream attractor. Populations typically exit the head attractor via both routes, splitting into two groups with distinct gene-expression trajectories (Fig 5A). We identified the NMP, PreNeural, and Early p3 ACs as head ACs of binary flip sub-landscapes, while the p2 attractor heads a related but slightly more complex architecture (discussed below). The binary decision from NMP to either neural or mesodermal fates [34,35] is a canonical example: LDA projection shows clear separation between the NMP, PreNeural, Early Mesoderm, and Mesoderm attractors, with transitioning cells forming bridges between populations (Fig 5A). Between D3 and D3.6, cells diverge from NMP along two trajectories—one leading to PreNeural and the other to Early Mesoderm (D3.6), which then transitions directly to Mesoderm at D4 (Fig 5B). The PreNeural decision behaves analogously: cells with high Foxa2 expression take the ventral route while those with high Pax6 and low Foxa2 expression take the intermediate route [36] (Fig A5 in S1 Appendix).

#### Transition analysis.

To validate the bifurcation structures and identify the genes involved in the transitions, we developed a gene expression analysis using tubular neighbourhoods along splines that approximate the escape-trajectory manifolds described above (Fig 5C-5D and Section A2 in S1 Appendix). Rather than relying on a single path connecting the source AC to a downstream AC, we repeatably subsample the transitioning cells, fit a spline approximating the mean pathway for the subsample, and compute the mean expression of the significantly varying genes within the corresponding tubular neighbourhoods along the spline. We then confirm that the resulting ensemble of time series produced in this way is reproducible across subsamples (Fig 5E) and in the case where branching routes are expected we demand these ensembles are internally consistent and clearly distinct from each other. We do this because cells are microheterogeneous (near-identical cells in or transitioning from a single AC are unlikely to have identical landscape) and therefore we require that all reasonable candidates for the unstable manifold have similar gene expression time series that are also clearly distinct from other routes. This approach detects genes that vary significantly during transitions - including genes that are not differentially expressed between ACs but have nontrivial transition dynamics, thereby identifying potential GRN components. In addition to the genes defining the effective gene space, we tracked approximately 150 curated transcription factors relevant to neural development.

To analyse branching cell state transitions from a given AC, we restrict attention to the cells within that AC and the neighbouring ACs to which they connect. As an illustration, the cells in Fig 5A suggest a potential branching from the NMP AC towards the PreNeural and Mesoderm ACs.

We apply two criteria to validate coherent temporal gene-expression patterns and to distinguish the proposed bifurcation from the alternatives. For the first criterion, we applied the subsampling and spline procedure described above, then verified that the resulting expression time series fell into two well-defined classes to which cells could be reliably assigned. For the NMP example, almost all relevant cells followed one of two well-defined pathways with clearly distinct temporal expression patterns (Fig 5C–5E). We also assessed whether the variation along the splines was consistent with the trajectories of a stochastic dynamical system: that is, whether the fluctuations were structured rather than dominated by excessive noise or high-frequency fluctuations. More details are given in Section A2.1, A2.3 in S1 Appendix.

For the second criterion we assess geometry of departure from the head AC (Box 2) to determine the architecture of the landscape. For example, while in a binary flip, cells leave along a shared initial direction before diverging, while in a binary choice, they depart in different directions from the outset. In practice, we assessed this by examining whether the gene-expression profiles of transitioning cells at the earliest exit stage form a single cluster (flip), or two separate clusters (choice) (Box 2).

To these criteria we later add further validation of the proposed bifurcation structure by fitting a quantitative model to the data.

This approach was used to validate binary flip architectures, including escape from a head attractor immediately before branching. For the NMP head attractor, it revealed upregulation of Nkx1.2 and Irx3 for escape along the PreNeural pathway and of Tbx6 and Foxc2 for the Mesoderm pathway (Fig 5E), providing support for a binary flip in this case.

The nature of the transition is further revealed by the temporal progression of cells along the unstable manifolds of the flip landscape (Fig 5F), which shows how the distributions of cells within the tubular neighbourhood advances along the manifold over time. Here we observe a pulse of cells leaving an AC seemingly in unison. The plot would look different if cells escape at a roughly constant rate via stochastic fluctuations: the distribution would then remain stationary rather than progressing, providing a way to distinguish the two cases. It also gives timing information: here the broader variance at later stages (e.g., at D3.4) supports the idea that cells experience delays and do not transition simultaneously.

The critical transition from ACs was further validated using the transition index of Mojtahedi and colleagues [16,61,62], which tends to rise sharply at exit (e.g., Fig 5G for exit from NMP). We applied the same analyses to the other branching sub-landscapes (Section A2.4 in S1 Appendix).

### The effect of changing Shh levels on the landscape

**Fig 6:**
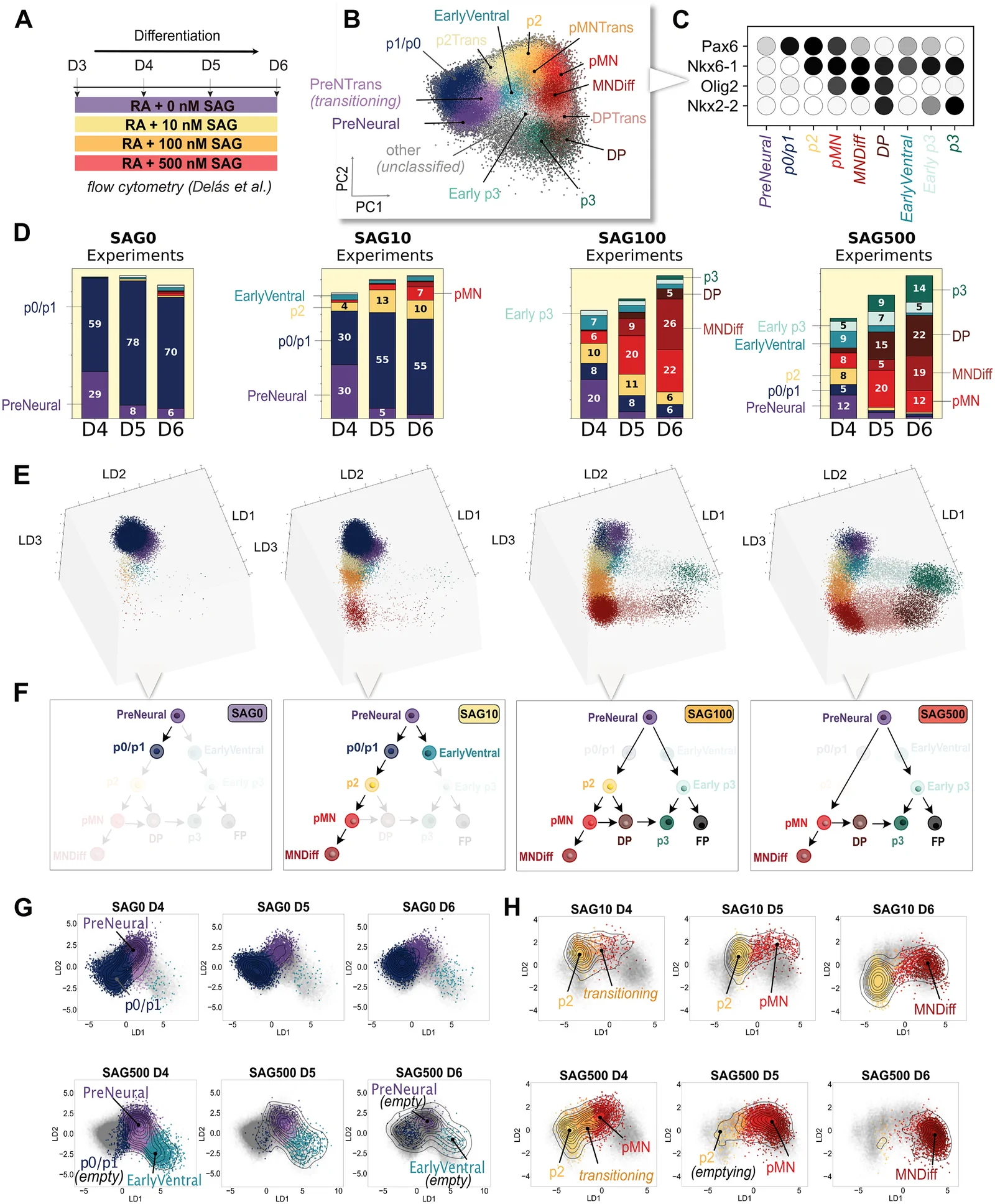
Flow cytometry analysis of cell states reveals SAG concentration-dependent neural progenitor specification. **A.** Schematic of the signalling conditions used for flow cytometry. **B.** 2D PCA projection of the ACs found using flow cytometry data. Data for all SAG concentrations and time points are combined. **C.** Dot plot indicating the expression of markers used to characterise the ACs in B (for the other clusters see Fig A8 B in S1 Appendix). The Floor Plate cluster (FP) was not identifiable in this dataset (see discussion in Section A4.2 in S1 Appendix). **D.** Percentage occupancy of the ACs as a function of Day (D) and SAG concentration (0, 10, 100, and 500 nM SAG). This shows the average proportions over 3 experimental replicates for 10 nM and 100 nM and 5 replicates for 0 and 500 nM (Section A3 in S1 Appendix). Blank spaces in bar plots correspond to the proportions of transitioning and unclassified cells, these are shown in Fig A8 C in S1 Appendix for all replicates. **E.** 3D representation in LDA coordinates of the clusters at all time points (unclassified cells excluded). At 0 nM SAG cells remain confined to the p0/p1 AC but start transitioning to p2 and pMN at 10 nM SAG. The ventral cell branch (green/cyan dots) that includes EarlyVentral, Early p3, and p3 only appears at high SAG concentrations (100, 500 nM). **F.** Schematic diagrams of attractors present at different SAG levels and the connections between them. This represents the consensus state because a landscape is associated with an individual cell, which are heterogeneous in state and signal. **G, H.** Temporal evolution of selected ACs (D4-D6) in LDA coordinates as a function of time and SAG concentration. Grey dots represent all cells in the group, coloured dots indicate cells present at the indicated time points with the colours indicating their cluster assignment. Contour lines indicate the density of cells at each time point. G. Binary flip decision from PreNeural to either p0/p1 or EarlyVentral. The figure shows 0, 500 nM SAG (see Fig A13 in S1 Appendix for other concentrations). (Top) The p0/p1 AC is stable when SAG is absent (0 nM) and there is no transition to EarlyVentral. (Bottom) The transition to EarlyVentral only appears at high SAG concentrations (100, 500 nM). PreNeural, p0/p1, and EarlyVentral empty over three days at the highest SAG level (500 nM). The attractors have bifurcated, but the unstable manifolds from PreNeural to pMN and Early p3 still pass through the regions in gene space formerly occupied by the bifurcated attractors. H. Direct transitions from p2 to pMN and from pMN to MNDiff. The figure shows 10, 500 nM SAG (see Fig A14 A in S1 Appendix for other concentrations). (Top) At 10 nM SAG, the p2 AC is relatively stable but shallow as cells can escape and transition to pMN over time. (Bottom) The same transition at 500 nM SAG where p2 has already bifurcated showing more rapid occupation of the pMN AC. The data underlying this Figure can be found at https://crick.figshare.com/projects/Neural_Tube_Decision_Landscape/250460 and at https://doi.org/10.5281/zenodo.15584010.

The route that a cell takes and its eventual fate depends critically upon the level of Shh signalling. Therefore, we set out to understand how Shh signalling shapes the decision landscape by creating or destroying attractors (fold bifurcations) or altering the transition routes between them (flip bifurcations). To this end, we identified five markers (Sox2, Pax6, Olig2, Nkx6.1, and Nkx2.2) sufficient to classify most ventral progenitor populations [36]. Using these markers, we generated three independent time resolved flow cytometry datasets using four SAG concentrations (0, 10, 100, and 500 nM) at days D4, D5, and D6 of differentiation (Fig 6A). We selected neural progenitors (Sox2^+^) using Gaussian Mixture Models (a statistical method that identifies overlapping sub-populations by fitting probability distributions) to identify clusters in the remaining 5-dimensional marker gene space [18] (Section A3 in S1 Appendix). Using statistical validation criteria analogous to those applied to the ACs from the scRNA-seq data (Section A3.3 in S1 Appendix) we identified the major progenitor populations (Fig 6B, 6C), analysed the local transitions between them, and tracked their proportions over time across SAG concentrations (Fig 6D). The proportions and the molecular composition for the transitioning populations for each experimental replicate are presented in Fig A8 B, A8 C in S1 Appendix. Unlike the scRNA-seq dataset, MNs and V3 neurones were not identified in the flow cytometry because only Sox2^+^ neural progenitors were selected for analysis.

Changes in SAG concentration resulted in different proportions of cell types and affected the topology of the connections in the landscape (Fig 6F). Although we can distinguish the Early p3 AC from the p3 AC, using the limited markers available we cannot distinguish FP, which is expected to co-express Sox2 and Nkx6.1 and to be present at D6. Nevertheless, the scRNA-seq data indicate it should be present at 500 nM SAG. We therefore included it as part of the schematic diagram in Fig 6F.

**Fig 7:**
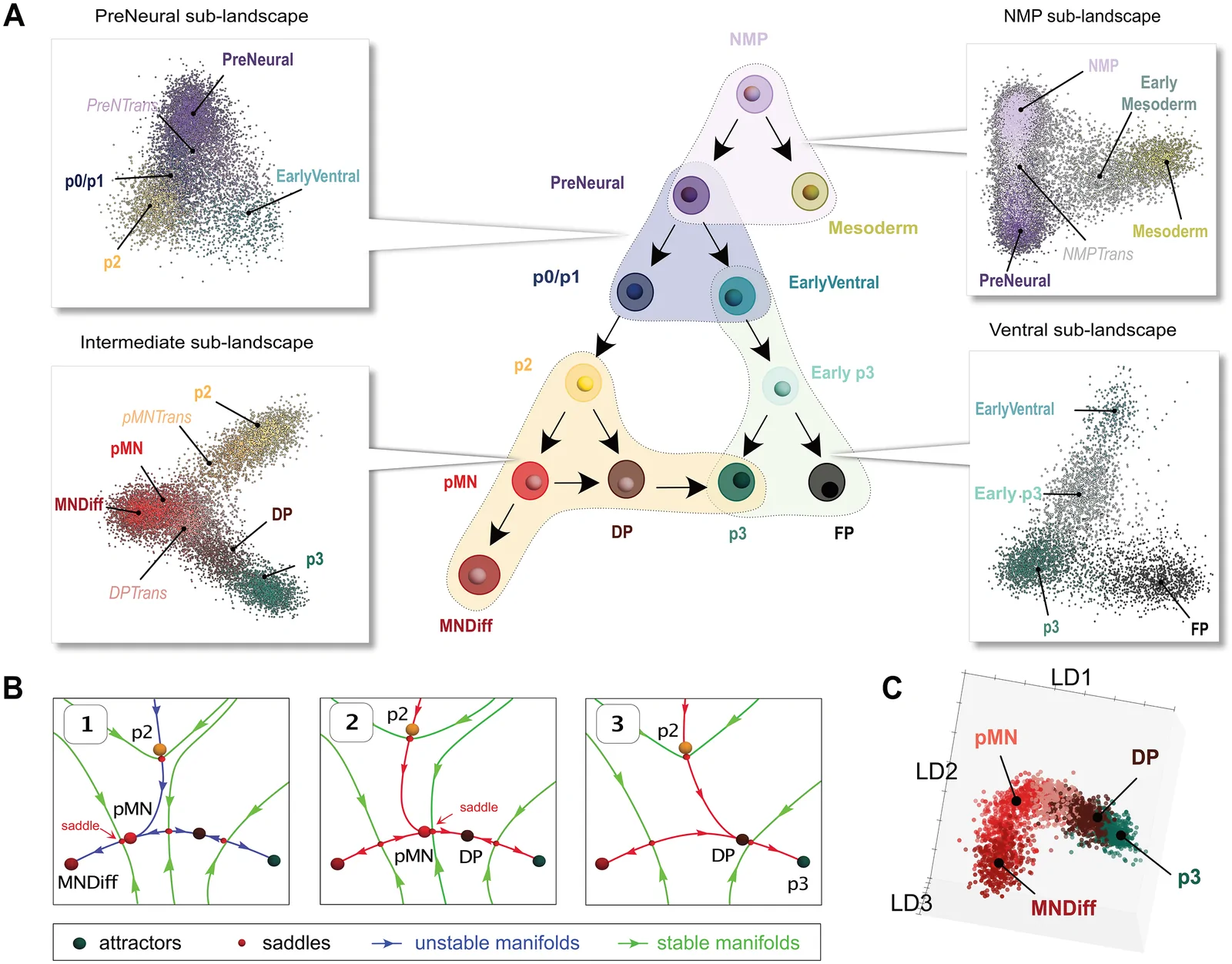
Sub-landscapes identification. **A.** Schematic diagram of all the possible transitions between cell states. A sub-landscape is defined as a group of neighbouring ACs and transitioning cells, projected in LDA space and highlighted on the diagram. A mathematical model should reproduce all the possible transitions. **B.** Distinct dynamical systems that model the transitions in the intermediate sublandscape. In Panel 1, p2 and pMN are close to their saddle and cells transition easily towards MNDiff. In Panel 2, the unstable manifold is directed towards DP and cells transition to DP via pMN. In Panel 3, the pMN attractor is bifurcated and DP is close to its right saddle, allowing a DP-to-p3 transition. **C.** The ACs MNDiff, pMN, DP and p3 form a smooth structure in LDA space, supporting our choice of using a linear landscape as in B to model the transitions from pMN to either MNDiff or DP. The data underlying this Figure can be found at https://doi.org/10.5281/zenodo.15584010.

This approach proved informative for revealing critical bifurcations that result from varying morphogen concentration (Fig 6E – 6H). A summary of all the ACs and transitions from the flow cytometry and scRNA-seq data is shown in Fig 7. The ventral branch comprising EarlyVentral, Early p3, and p3 states emerged from PreNeural only at the higher SAG concentrations, indicating the occurrence of a flip bifurcation (Fig 6G) as verified in the scRNA-seq analysis (Fig A5 in S1 Appendix).

The AC containing exclusively Pax6^+^ cells, labelled p0/p1, captures most cells at 0–10 nM SAG (Fig 6G). But, at higher concentrations, Nkx6.1 and Olig2 levels rapidly rise within the Pax6^+^ cells, leading to the emergence of p2 and pMN (Fig 6H).

While the p2 AC is present and stable at 10 nM SAG, it destabilises at higher SAG concentrations, with these cells instead adopting a pMN identity through up-regulation of Olig2 (Fig 6H) or a DP identity through up-regulation of Olig2 and Nkx2.2 (Fig A14 F in S1 Appendix). The pMN AC is populated by a few cells at 10 nM SAG but increased occupation of pMN and MNDiff is apparent at 100–500 nM SAG. By contrast, DP and p3, both expressing Nkx2.2, required a concentration above 100 nM SAG to appear (Fig 6D – 6F).

The time-resolved flow cytometry data confirmed the transition routes identified in the scRNA-seq data and revealed SAG-concentration dependent bifurcations (all remaining transitions are presented in Section A4 in S1 Appendix in particular Figs A13-A14 in S1 Appendix). Thus, by combining transcriptional profiling with protein-level measurements, we established a comprehensive map of stable cell states and their connecting transition paths as a function of SAG concentration (Fig 7A).

### Mapping the complete neural progenitor landscape

The unstable manifold transition analysis proved robust to different projections and data modalities, capturing the progression of cell fate during all neural progenitor transitions (Section A2 in S1 Appendix). Gene expression changes along transition routes found in both modalities revealed consistent patterns. Exit from a head AC is marked by the down-regulation of genes that define this AC and the simultaneous up-regulation of markers associated with the target ACs, consistent with proposed multi-lineage priming mechanisms of fate commitment—the phenomenon in which cells simultaneously express low levels of genes associated with multiple potential fates before committing to a single lineage [63].

Together, the method captured the gradual gene expression changes during transitions and identified route-specific gene expression programs. This provided evidence for well-defined transition routes and critical fate decision points essential for a quantitative model of the developmental landscape.

Overall, we identified four sub-landscapes comprising ACs and transitioning cells, which we called respectively *NMP*, *PreNeural*, *intermediate* and *ventral* sub-landscapes (Fig 7). We analysed each of these separately, following the approach used for the NMP sub-landscape. Each of the branching decisions shown, except those in the intermediate sub-landscape, is a binary flip bifurcation with exit paths selecting between the downstream states shown. The evidence for this is given in Section A2 in S1 Appendix.

The decisions in the intermediate sub-landscape are particularly interesting. When the p2 AC is present (i.e., when the SAG level is 10 nM or 100 nM) it is a head attractor of this sub-landscape (Figs 7B and A6 in S1 Appendix). Cells exiting p2 proceed either to the pMN or DP ACs. We identified the transition as a binary flip: cells exiting p2 fall into two distinct expression classes, one up-regulating Olig2 and Neurog2 along the pMN route and the other showing coordinated up-regulation of Olig2, Hes1, Rfx4 and Nkx2.9 towards DP, and at the earliest exit stage these cells form a single cluster (pMNTrans), departing along a shared direction before diverging—the geometric signature of a flip rather than a choice (Box 2). The pMN-specified cells then progressed through a Neurog2-high MNDiff intermediate before terminal MN differentiation [44]. pMN and DP are themselves part of a linear structure of four ACs—MNDiff, pMN, DP and p3—with saddles between each (Fig 7B, 7C). We observed the progressive loss of DP cells by D7 with convergence towards p3, supporting a direct DP-to-p3 transition (Fig A7 B in S1 Appendix), which we confirmed by flow cytometry (discussed below). Lineage studies agree, since a subset of p3 progenitors descend from cells with an Olig2 expression history while FP cells rarely do [64]. Cells transitioning from pMN form two distinct, nonconnected clusters (DPTrans and MNDiff)—one up-regulating Nkx2.9 along the DP route and the other Neurog2 towards MNs—rather than a single cluster co-expressing both markers as multilineage priming would produce in a flip. We therefore identify the pMN decision as a binary choice rather than a flip (Box 2). Together, the transitions pMN-to-DP, pMN-to-MNDiff and DP-to-p3 imply three saddles whose 1-dimensional unstable manifolds connect each pair; the data show these attractors and saddles lie close together (Box 2), so the three pathways link smoothly into the linear structure of Fig 7B. We analyse the gene expression along the approximate unstable manifolds in Fig A6 D-A6 E in S1 Appendix), and in later sections fit a mathematical model capturing this more complex bifurcation.

### Constructing a landscape model

We next developed a mathematical model of the observed decision-making dynamics in response to different Shh signalling levels. Rather than modelling gene-gene interactions as in conventional GRN models, our approach constructs a quantitative model of the cell fate decisions at the population level including the bifurcation structure underlying these decisions. This model can generate experimentally testable predictions because it can be used to simulate unseen situations such as changing morphogen conditions and missing time points. The model consists of deterministic differential equations, with parameters dependent on SAG concentration, and a space-dependent stochastic component.

The key step is the construction of the deterministic model component as this drives the decision-making. We partitioned the system into overlapping local sub-landscapes corresponding to the different transition architectures identified above (Fig 9). Because each sub-landscape corresponds to a specific transition type, catastrophe and bifurcation theory [65] can be used to provide a standardised minimal set of equations—called a *normal form*—that captures the essential structure of the signal-dependent decision dynamics within it. Each normal form contains a small number of parameters with clear interpretations that fully reproduce the effects of the potential signals changing the landscape. In particular, some govern when a stable state is destroyed by a fold bifurcation, others determine which of two downstream fates is favoured at a flip bifurcation. The use of these normal forms enables systematic understanding of how the parameters, which represent the effect of signalling, affect cell fate decisions. This provides precise control over which parameter drives each bifurcation type. Details of how the normal forms are chosen can be found in Section B2.5 in S2 Appendix and the nature of the bifurcation sets for the normal forms is discussed in Box 3.

The parameters function phenomenologically rather than representing specific biological rates and can be regarded as combinations or functions of the underlying biological parameters of the relevant GRN. They act as control variables that determine the system’s critical decision points. Thus bifurcation sets clarify how signalling levels translate into decision outcomes and reveal how population heterogeneity propagates through the decision landscape to generate outcome variability.

For systems of the sort we are studying it has been shown that with the local dynamics the possible global topologies of the landscapes (i.e., the landscape graphs) are independent of the number of genes involved [17]. It is therefore reasonable that, for our system, a two-dimensional representation can capture the essential bifurcation structure and transition pathways present in the full high-dimensional gene expression space.

**Box 3. A dynamical-systems view of cellular decision-making: bifurcation sets and decision heterogeneity. Terms explained: bifurcation curves and bifurcation set, fold–flip points, head attractor, cusp bifurcation, bistability.**

In constructing the global dynamical landscape, we decomposed it into a collection of sub-landscapes. The bifurcations of the full system can then be understood in terms of the bifurcations within each sub-landscape. In our setting, each sub-landscape depends effectively on two parameters, and so the qualitative behaviour is organised by the generic bifurcations of two-parameter systems.

**Two-parameter structure and decision boundaries.** The parameter space (corresponding to signal values) is therefore two-dimensional, and can be visualised as a plane in which each point represents a specific signalling environment. Changes in signals correspond to movement of this point through parameter space.

Bifurcations occur when this point crosses either a fold curve or a flip curve, along which fold and flip bifurcations occur respectively and these curves make up the *bifurcation set*. These curves are smooth—a curve with no sharp corners or abrupt changes in direction—except at two types of special organising points occurring at isolated locations along them (cusps and fold-flip points). The bifurcation set partitions the parameter space into regions within which the system has the same set of stable cell states and the same transition paths between them.

**Fig 8:**
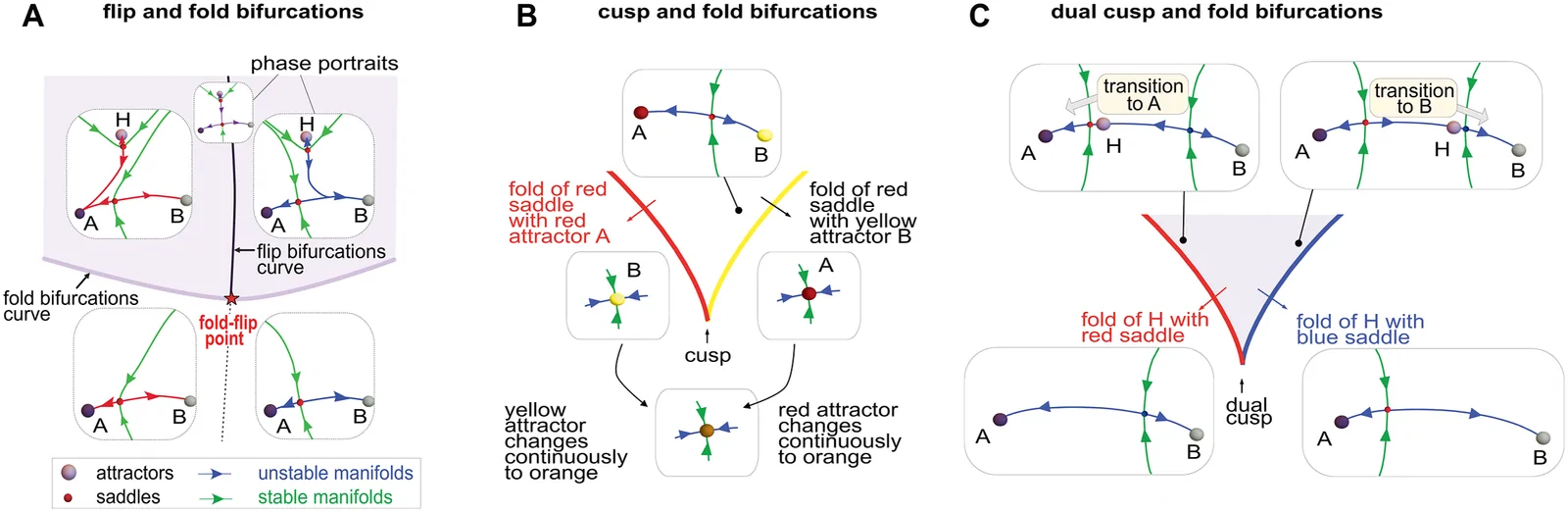
Bifurcation curves divide up the parameter space into regions and in each region the qualitative form of the dynamics (phase portraits) is shown. Blue curves are the unstable manifolds of the saddles (small red balls) and the green curves are stable manifolds. **A.** Dynamics around a fold-flip point (red asterisk). **B.** Fold bifurcation curves meeting at a standard cusp. **C.** Fold bifurcation curves meeting at a dual cusp. After bifurcation cells transition from the central attractor H to one of the two peripheral ones (A or B).

Leaving a region by crossing a fold curve changes the number of stable states through the creation or annihilation of an attractor–saddle pair, while crossing a flip curve changes the transition paths of the landscape without altering the number of stable states (Fig 8A).

**Fold–flip points and branching decisions.** Flip curves generically terminate on fold curves at special points known as *fold–flip points* (Fig 8A). These points organise the local structure of decision-making. In their neighbourhood, parameter space is divided into three regions: two regions in which three stable states *H*, *A*, *B* coexist, separated by a flip curve, and a third region in which only two stable states *A*, *B* remain, following the loss of the intermediate one *H* in a fold bifurcation (Fig 8A).

Our theory predicts that, at a branching point, cells pass through such an intermediate (“head”) attractor *H*. Cells may either remain near this until a signal-driven bifurcation occurs, or, when it is weakly stable, linger near it before escaping via stochastic fluctuations. If, following the loss of *H*, cells can proceed to either fate *A* or *B*, this indicates that the underlying fold bifurcation occurs close to a fold–flip point (Fig 8A).

**Cusps and bistability.** Fold curves can develop isolated codimension-two points called *cusps*, where the curve folds back on itself. At a standard cusp (Fig 8B), a saddle interacts simultaneously with two attractors, organising regions of *bistability* in parameter space. Within these regions, two alternative fates coexist, and small changes in signals can switch which fate is realised. At a dual cusp (Fig 8C) an attractor interacts simultaneously with two saddles. This is the basis of the *binary choice landscape*.

**Implications for developmental decisions.** The structure of bifurcation sets fundamentally shapes how molecular heterogeneity is translated into cell fate outcomes. When noise levels are low, cells with very similar molecular states can nevertheless adopt different fates if they lie on opposite sides of a bifurcation curve. Thus, heterogeneity in differentiation outcomes need not arise from intrinsic stochasticity, but can instead reflect a population distributed across decision boundaries in parameter space (e.g., Fig 11F–11G below). Understanding the geometry of these bifurcation sets, and how experimentally inferred parameters map onto them, is therefore essential for interpreting decision heterogeneity in single-cell data.

#### Connecting the sub-landscapes to obtain a global model.

**Fig 9:**
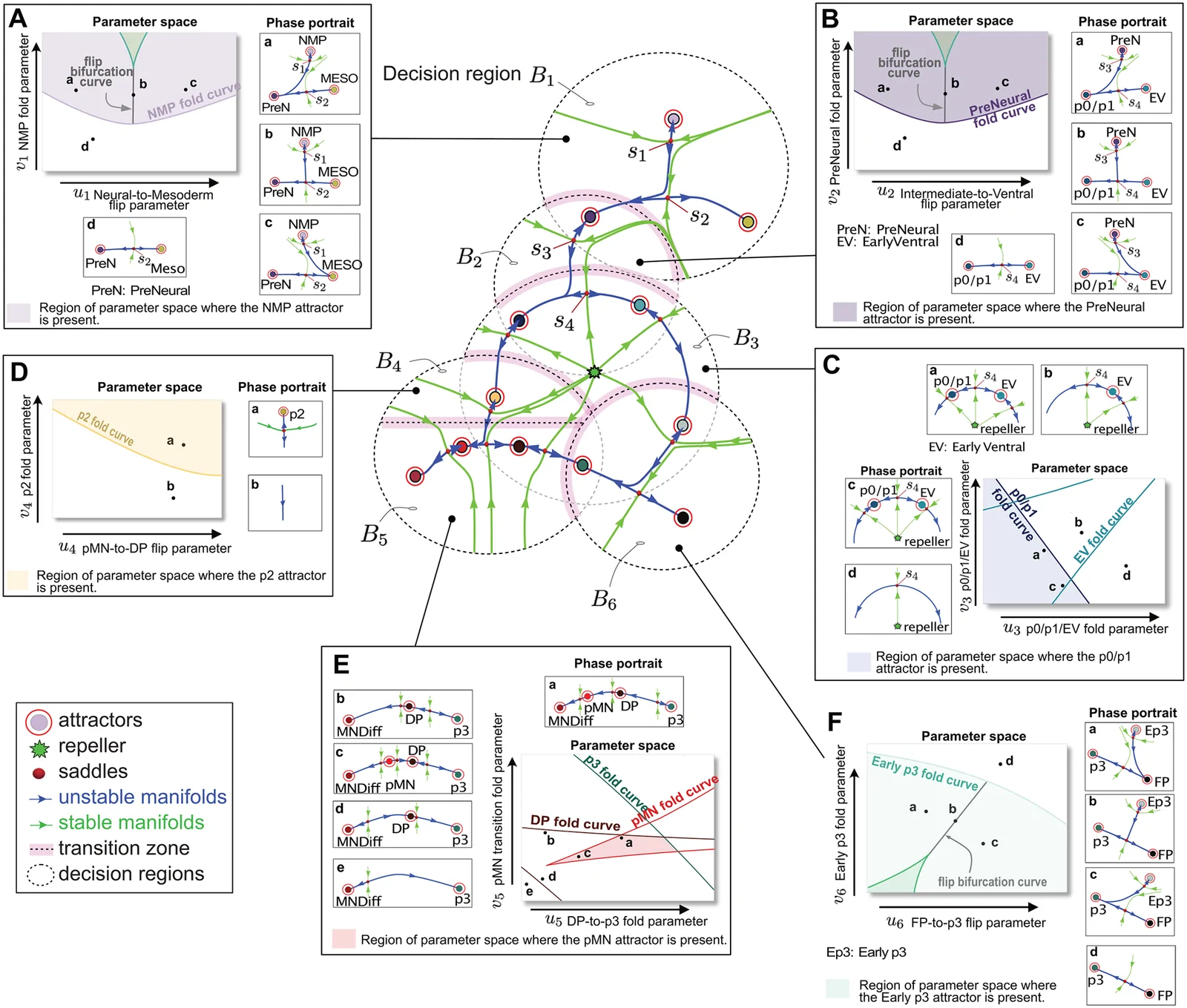
Constructing the full landscape model. The sub-landscapes used to construct the model showing one realisation of the unstable manifolds. Changing parameters allows unstable manifold flips or the bifurcation of attractors. The pink regions (transition zones) show where bump functions were used to glue the landscapes together. These divide the phase space into 6 domains and as the state crosses a transition zone its dynamics transition smoothly to the dynamical system governing the regions it has entered into. The central repeller visible in the phase portrait of region *B*_3_ is a necessary consequence of the landscape construction. In higher-dimensional state space it would correspond to an index-2 saddle (a fixed point with a 2-dimensional unstable manifold). **A–F.** Each panel shows the parameterised dynamics in the decision regions $B_{1}-B_{6}$. These indicate the form of the bifurcation set which divides the parameter space into regions where the qualitative form of the dynamics is constant. They also show the configuration of the dynamics in each of these regions. As a bifurcation curve is crossed either a flip bifurcation occurs or one of the attractors appears or disappears (fold bifurcation). **A.** 2D Parameter space of the NMP sub-landscape with a fold parameter (y-axis) that controls the bifurcation of the NMP attractor and a flip parameter (x-axis) that controls the flip bifurcation from neural fates to mesodermal fates. **B.** Similar to A for the PreNeural sub-landscape. In this case the flip parameter controls the transition from intermediate (p0-p2-pMN) to ventral fates (EarlyVentral, Early p3, p3 and FP). **C.** Parameters that control the fold bifurcations of the p0/p1 and EarlyVentral attractors. **D.** Parameters that control the fold bifurcation of the p2 attractor. **E.** The dynamics of the MNDiff, pMN, DP and p3 attractors. The bifurcation set consists of 2-fold curves for the DP attractor (brown) meeting at a dual cusp (not shown) and two curves of fold points controlling the bifurcation of the pMN attractor (red), also meeting at a dual cusp (shown). This bifurcation structure is motivated by the experimental observation that cells can differentiate to motor neurones from pMN as well as transitioning to a DP fate by up-regulating Nkx2.9 and Hes1. This means that the saddles on both sides of the pMN attractor must interact with it. **F.** Parameters controlling the fold bifurcations of the Early p3 attractor and flips of the unstable manifold of the saddle near the Early p3 attractor that leads cells to either p3 or FP fates. The bifurcation set consists of a fold curve and a flip curve that meets the fold curve at a fold-flip point.

The normal form models for the sub-landscapes are then connected using the simple topological relationships deduced from the data, with connections occurring along transition routes that avoid attractors and bifurcation-critical regions (Figs 9 centre and 10A). This design enables the sub-landscapes to be joined using ideas from differential topology [66] (Section B2.4 in S2 Appendix) for joining local systems together to produce a global one. The result is a dynamical system that depends smoothly on its parameters (Section B2.5 in S2 Appendix). The model qualitatively captures the key bifurcations observed in the experimental data, including fold and flip bifurcations.

**Fig 10:**
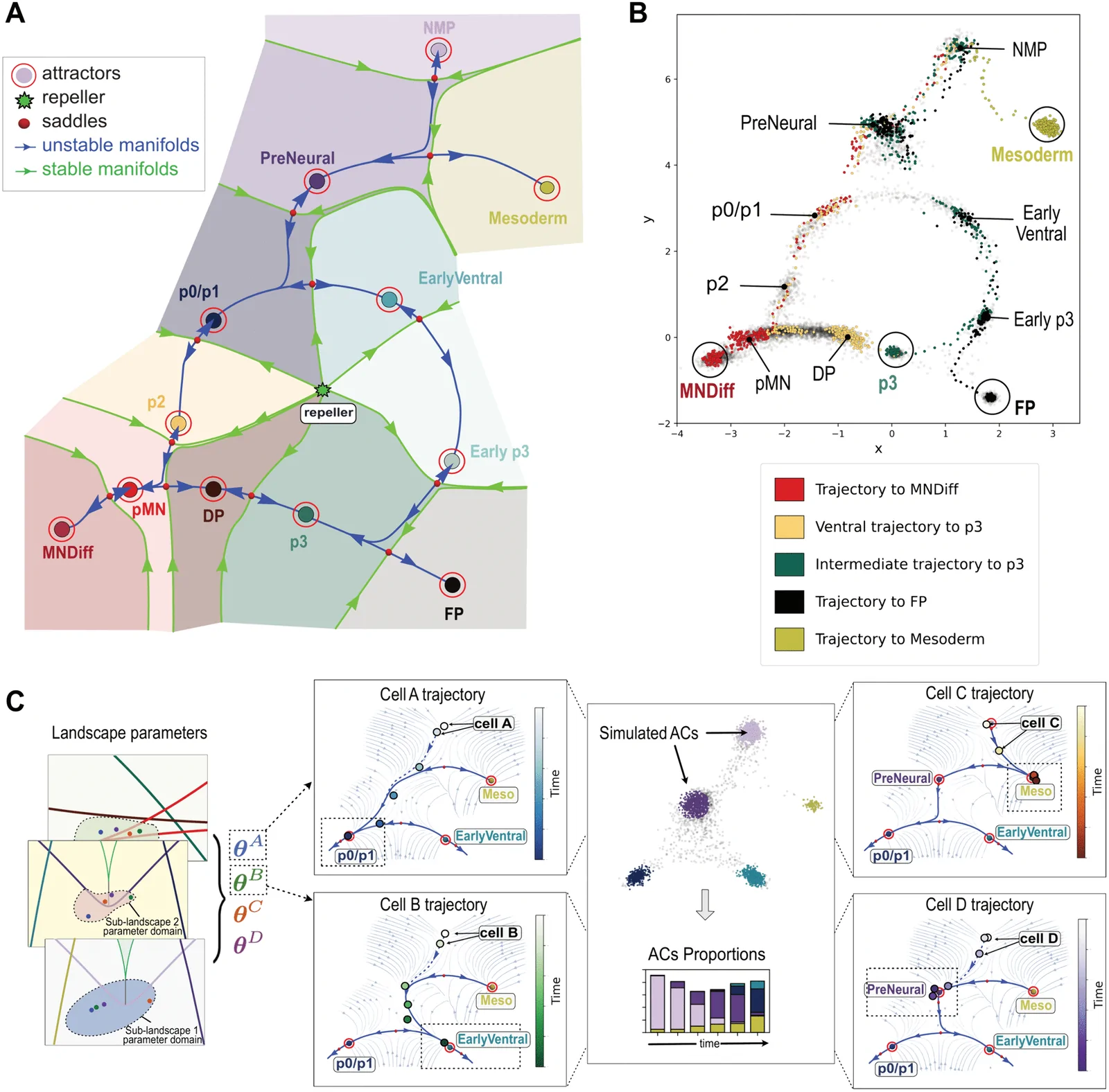
Global landscape simulation. **A.** Phase portrait of the global landscape for a fixed set of parameters when all attractors are present. The blue and green curves are respectively the unstable and stable manifolds of the various saddles. The green stable manifolds of the saddles separate the basins of attraction of the various attractors which are coloured accordingly. If the state of a reprogrammed cell falls in one of these coloured domains, it will converge towards the corresponding attractor and adopt that specific fate. We arbitrarily fixed a direction for each unstable manifold. B. Five stochastic single-cell trajectories followed for 400 simulation time steps coloured according to the eventual fate. Grey dots indicate other cell trajectories, included to help visualise the shape of the landscape. Notice that while pseudo-time makes sense for the transitioning segments of a trajectory, it does not make sense for a full trajectory because cells spend a random period of time near attractors. **C.** Schematic of the simulation procedure, illustrated for the first two binary decisions. For each cell μ, parameters for the individual sub-landscapes are selected within their respective parameter domains (two components in each 2D bifurcation panel represented by a dot coloured differently for each cell). Together, they form the components of a parameter vector $\theta^{\mu }$ of the global landscape (illustrated here for 4 cells labelled A, B, C and D with parameters $\theta^{A}$, $\theta^{B}$, $\theta^{C}$, $\theta^{D}$). These parameters define the configuration of attractors, saddles and unstable manifolds of the cell’s deterministic dynamics. Adding stochastic fluctuations to these dynamics, we simulate a stochastic trajectory for each cell, recording their positions in state space at discrete time points. Each of the panels on the left shows the deterministic (local) landscape for each cell determined by their respective parameters. In each panel, the evolving cell is coloured according to its time step. Different sets of parameters produce different landscape models resulting in distinct possible fates for each cell (dashed rectangles in each panel). If an attractor is present in a cell’s landscape (e.g., PreNeural for cell D), the cell may remain trapped in this attractor as we see for the purple dots, indicating the position of the cell over time, staying near the PreNeural attractor. Expanding the parameter sampling allows the simulation of a large number of cells (central panel). Clustering the simulated cells identifies ACs (coloured according to the cell state they correspond to), and the proportion of cells in each AC at each time points were compared to the experimental data.

#### Dynamical stochasticity and cell-dependent dynamics.

To model the stochastic nature of the dynamics due to intrinsic noise arising from the randomness of biochemical reactions within a cell we include a spatially dependent diffusion term setting the noise level as an additional parameter. We allowed the noise to vary between the various regions of phase space, accounting for transition-specific noise variability (Section B3.1 in S2 Appendix). Finally, velocity parameters are included that allow the speed along trajectories to vary from region to region. This provides a highly flexible but minimally parameterised model that can accurately capture the behaviour in each local sub-landscape.

As mentioned above when discussing microheterogeneity, nongenetic variability, arising from within-cell processes such as differences in cell size, ribosome number etc, play a role in determining cell fates. To account for this nongenetic variability, we allow for variation in each cell’s parameters producing a distinct dynamic for each cell (Fig 10B, 10C).

When we combine these various types of stochasticity we observe dynamics where cells starting in identical states can traverse widely different developmental trajectories (Fig 10B). Moreover, the timing with which cells follow the same trajectories tends to be highly variable because cells can remain trapped in shallow attractors for extended periods before escaping due to stochastic fluctuations, and then progressing relatively linearly along the unstable manifolds (Fig 10C). We quantify this variation in timing below in Fig 13D when we discuss the DP to p3 transition, showing that it can be very large and represents a challenge to the notion of pseudotime.

### Fitting the model to experimental data

We estimated model parameters for each SAG concentration using Approximate Bayesian Computation particle filtering [15,18,67–71] which yields a probability distribution over plausible parameter values rather than a single best-fit estimate. This involves simulating each cell’s trajectory using the stochastic model on a finite time interval, calculating the proportions of simulated cells forming a *simulated AC* at regular intervals, and comparing these with their experimental counterpart (Fig 10C). Parameters are iteratively sampled until convergence to a *posterior parameter distribution* (PPD) that optimally reproduces the experimental cell state proportions. Details are provided in S2 Appendix, Section B4 for the general fitting procedure, Section B5 for the fitting to the flow cytometry data, and Section B7 for the scRNA-seq data.

**Fig 11:**
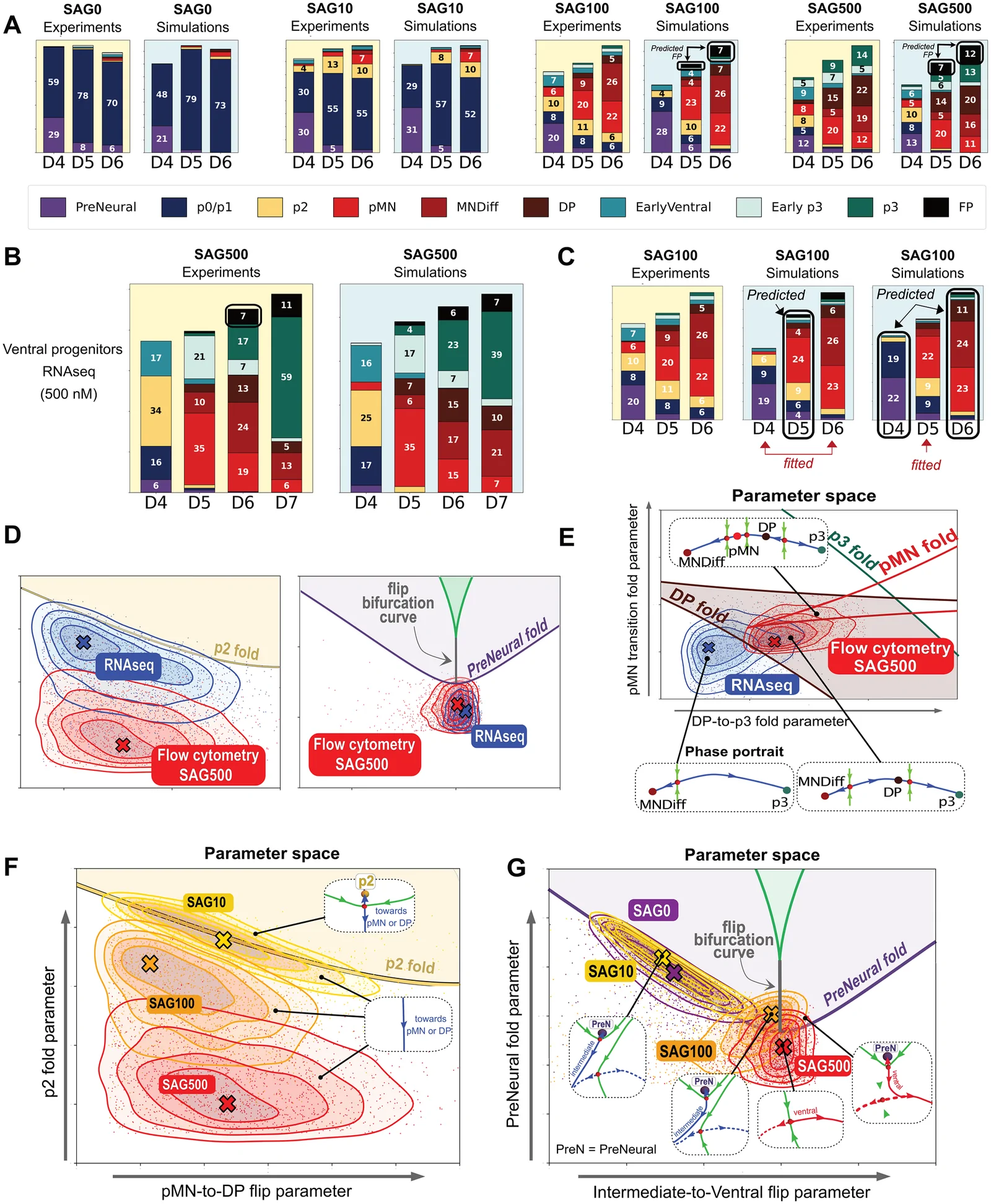
Parameter estimation and bifurcation analysis of the dynamic landscape model. **A.** Experimental proportions (yellow background) compared to the simulated proportions (blue background) after parameter fitting for each SAG concentration using the flow cytometry data. The experimental proportions were obtained by averaging the proportions for 3 individual experimental series for 10 nM SAG, 100 nM SAG and for 5 experimental series for 0 and 500 nM SAG (Section A3 in S1 Appendix). The predicted FP proportions (12%) obtained from simulations at 500 nM SAG are consistent with those (7%) observed in the scRNA-seq at the same SAG concentration (see B below). **B.** Similar to A for the scRNA-seq data at 500 nM SAG. **C.** (Left) Experimental proportions for flow cytometry 100 nM SAG. (Middle) Fitting the model to only the D4 and D6 experimental proportions accurately predicts the intermediate D5 proportions. (Right) Fitting the model to only the D5 experimental proportions predicts the proportions at D4 an D6. **D–G.** Dots represent parameters accepted by the Approximate Bayesian Computation particle filter. Contour lines are used to estimate the PPDs projected on the corresponding sub-landscape parameter space (blue: 500 nM SAG scRNA-seq, red: 500 nM SAG flow cytometry, orange: 100 nM SAG flow cytometry, yellow: 10 nM SAG flow cytometry, purple: 0 nM flow cytometry). The other curves show the bifurcation set where fold and flip bifurcations occur for the different sub-landscapes. Panels (E–G.) contain the sub-landscape phase portraits that arise for parameters chosen within each region of bifurcation set. **D.** (Left) p2 sub-landscape bifurcation set. (Right) PreNeural sub-landscape bifurcation set. The flow cytometry and scRNA-seq PPDs at 500 nM SAG fall in the same regions of parameter space, delimited by fold and flip curves. The associated dynamics and decisions are thus qualitatively identical. **E.** Intermediate sub-landscape bifurcation set with the flow cytometry and scRNA-seq PPDs at 500 nM SAG. The shaded region indicates the set of parameters for which the DP attractor is present. As the blue PPD overlaps the DP fold bifurcations curve, we observe heterogeneity: some cells remain trapped in the DP attractor whereas others rapidly transition to p3. The flow cytometry PPD (red) remains largely within the shaded region, where the DP attractor is present, although the fraction of cells with parameters close to the DP fold curve still transition from DP to p3 due to stochastic fluctuations. **F.** Bifurcation set of the sub-landscape controlling the p2 transition. The yellow fold curve represents the parameter values where the p2 attractor bifurcates. At 10 nM SAG, the PPD lies on both sides of the p2 fold curve, suggesting that the p2 attractor is present, but shallow, for half of the cells and bifurcated for the other half in which case cells transition to a downstream attractor. At higher SAG concentrations (100, 500 nM), the PPDs lie outside the shaded region suggesting that the p2 attractor is bifurcated for all cells. The 0 nM PPD (purple) is not shown, as the parameters did not converge due to the absence of p2 cells at this SAG concentration (the parameters are distributed across the entire region). **G**. Bifurcation set of the sub-landscape governing the binary decision from PreNeural to intermediate or ventral fates. Parameters on either side of the grey flip curve yield distinct sub-landscapes: one with an unstable manifold (blue) connecting PreNeural to an intermediate fate (p0/p1, p2, pMN), and another (red) to a ventral fate (EarlyVentral, Early p3, p3, FP). Crossing the purple fold curve causes the PreNeural attractor to bifurcate, with fate determined by the basin of attraction the cell is in. At low SAG concentrations (0–10 nM), PPDs lie left of the flip curve, indicating transitions primarily towards intermediate fates. At higher concentrations (100–500 nM), PPDs are near the fold–flip point, allowing heterogeneous transitions to both intermediate and ventral fates. The data underlying this Figure can be found at https://crick.figshare.com/projects/Neural_Tube_Decision_Landscape/250460 and at https://doi.org/10.5281/zenodo.15584010.

Fitting the model to the flow cytometry data for all SAG levels (Fig 11A) and the scRNA-seq data for SAG 500 nM (Fig 11B) gave good agreement with the experimental proportions of the ventral progenitors. FP cells could not be clearly distinguished in the flow cytometry data but the model provided an estimate (12%) of FP proportions at D6 for SAG 500 nM which is consistent with those observed in the scRNA-seq data (7%) in Fig 11B. Additional validation using flow cytometry is given Fig B9 B in S2 Appendix. Importantly, we observed that fitting on a limited set of time points still enabled accurate prediction of cell state proportions at intermediate time points (Fig 11C).

#### Comparison between flow cytometry and scRNA-seq.

Given longstanding questions about the relationship between expression levels of mRNA and protein measurements, it was unclear whether model parameters fitted to both types of data would agree. We therefore compared the PPDs obtained by fitting the model to the scRNA-seq data with those of the flow cytometry at 500 nM SAG by projecting them onto the bifurcation sets for each sub-landscape to reveal how signalling conditions control cell fate. We define two PPDs as qualitatively the same if they occupy the same region of the bifurcation set delimited by fold and flip curves. For most sub-landscapes, the SAG 500 nM PPDs were qualitatively the same (Fig 11D), This agreement additionally validates the robustness of the landscape structure across measurement modalities.

There was, however, one notable difference. The scRNA-seq PPD crossed the DP fold curve while the cytometry PPD did not (Fig 11E), forcing DP cells to rapidly transition to p3. We hypothesised that this discrepancy reflected the substantially increased number of p3 cells in the scRNA-seq data on D7 (Fig 11B), as these include the V3 neurones that become abundant at D7. In comparison, the flow cytometry data extended only to D6 and the numbers of p3 cells were lower and did not include V3 neurones as only Sox2-expressing cells were selected. Simulations revealed that the substantial p3 population at late time points cannot be achieved solely through the PreNeural-to-p3 pathway via the ventral sub-landscape. This suggested that the PPD crossing the DP fold curve (Fig 11E) reflects an alternative route: direct transition from DP to p3. The model predicts that cells can reach the p3 fate through two distinct developmental pathways, a prediction we validate below.

#### Bifurcation sets determine how molecular heterogeneity translates into decision heterogeneity.

As discussed in Box 3, bifurcation sets fundamentally shape how molecular heterogeneity affects cell fate outcomes. When noise levels are low, two cells, whose landscape parameters are in the same region of the bifurcation set, will make the same decisions with high probability. Conversely, nearly identical cells can make reliably different fate choices if a bifurcation curve divides them into distinct regions. This implies that heterogeneity in differentiation outcomes among molecularly similar cells arises from a cell population straddling bifurcation boundaries rather than from intrinsic noise (Fig 1B). Understanding bifurcation set structure and how estimated parameters map onto it is therefore essential for interpreting decision heterogeneity in single-cell data. We analysed the four PPDs obtained by fitting the model to flow cytometry data at constant SAG concentrations.

The bifurcation set controlling the direct transition from p2 contains a fold bifurcation curve (Fig 11F). Crossing this curve destabilises the p2 attractor, forcing a cell to transition to pMN or DP. At 10 nM SAG, parameters fall on both sides of the fold curve, creating heterogeneity where some cells remain in p2 while others transition. This reproduces the bistability in the p2 and the pMN cells population observed at D6 in Fig 6H. At higher concentrations (100, 500 nM), the PPDs lie below the fold curve, indicating that the p2 attractor has bifurcated for all cells and driving uniform differentiation to pMN. Even at low SAG concentration (10 nM), this in vitro protocol fails to stabilise the p2 state. The model predicts that producing a persistent population of p2 cells is not possible by modulating Shh signalling alone.

In the PreNeural sub-landscape bifurcation set, a flip and a fold curve intersect at a fold-flip point (Fig 11G). At low SAG (0, 10 nM), the PPDs localise to the left of the flip bifurcation curve, biasing cells to intermediate fates. At high SAG (100, 500 nM), the PPDs concentrate near the fold-flip point, generating heterogeneous outcomes between the two fates.

Other informative examples showing the relationship between bifurcation and PPDs mappings and validating agreement between scRNA-seq and flow cytometry data are discussed in Sections B5.1 and B7.3 in S2 Appendix).

Overall, the fitted model accurately captured the experimental dynamics (S1–S3 Videos), and successfully reproduced the observed bifurcation behaviours. This framework revealed how individual bifurcations combine to generate complex decision structures and provides a qualitative and quantitative explanation for Shh signalling dependent patterning of ventral neural tissue and makes testable predictions.

#### Model predictions of signalling induced landscape changes.

We used the fitted model to develop and test various hypotheses. We first asked whether temporal changes in Shh signalling resulted in instantaneous or delayed landscape reconfigurations. We focussed on three principal decision points: the transition from PreNeural to either the intermediate or ventral sub-landscapes; the transition from p0/p1 to p2 that leads to pMN; and the connection from DP to p3 responsible for the circular topology of the landscape.

**Fig 12:**
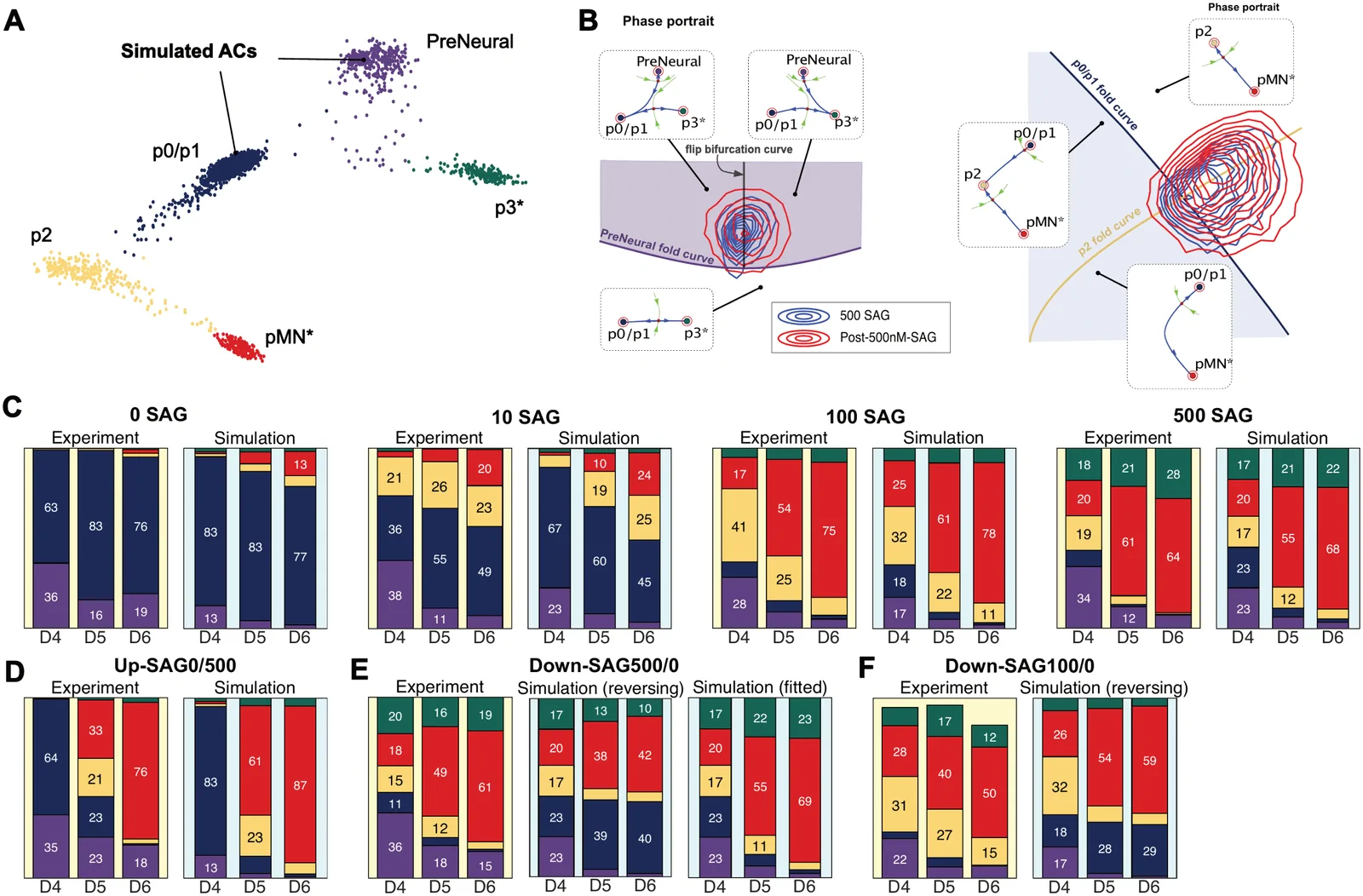
Experimental validation of model predictions by signalling perturbations. **A.** Simulated data showing the attractors and transition routes. Dots represent simulated cells retained at 3 time points (D4, D5, D6). These are aggregated into simulated ACs coloured by the cell state to which they correspond. **B.** PPDs from model fitting for the 500 nM SAG condition (blue) and the post-high-SAG condition (red) are close to identical, revealing that the two conditions are approximately equivalent. (Left) Projection of the PPDs on the bifurcation set of the binary flip sub-landscape. (Right) Projection of the PPDs on the bifurcation set of the linear sub-landscape. **C.** Comparison of average simulation results (blue background) for the constant SAG conditions with the corresponding experimental results (yellow background). Results are average over 10,000 simulated proportions using parameters drawn from the fitted PPDs (Section B8 in S2 Appendix). **D.** As C but for the Up-SAG0/500 condition. The prediction aligns with the experimental data. It shows that delaying SAG application by 24 h commits the cells to differentiate into pMN${\;}^{*}$ rather than p3${\;}^{*}$. **E.** As C but for the Down-SAG500/0 condition, modified as follows: (middle) uses the fitted PPDs (500 and 0 nM SAG) obtained from C and assume instantaneous response to signal change, (right) uses the refitted PPD. The latter shows better agreement with the experimental results demonstrating a lack of response to the change of signal. **F.** As for the first two panels of E for the Down-SAG100/0 condition where the fitted PPDs (100 and 0 nM SAG) obtained from C are used. We observe the same phenomenon as in E (middle panel), despite lower levels of the signal. The data underlying this Figure can be found at https://crick.figshare.com/projects/Neural_Tube_Decision_Landscape/250460.

To examine the first two decisions, we recast the model into a simplified version that focussed on these transitions (Fig 12A). This reduced model aggregated several related cell states (combining NMP/PreNeural, EarlyVentral/Early p3/p3, and pMN/MNDiff/DP) while preserving the essential topology of a binary flip landscape followed by a linear transition pathway (Section B8 in S2 Appendix). In this framework, the ventral sub-landscape consists of a single state p3*, and the intermediate sub-landscape comprises two states: p2 and the combined pMN/MNDiff/DP state, relabelled pMN*. The model accurately reproduced the proportions of cell states observed in various SAG concentrations (Fig 12C). The full global landscape model produces equivalent predictions for these signalling perturbation experiments; details are provided in Section B6 in S2 Appendix. The simplified version is presented here.

**Timed pulses.** We used the model to simulate the effect of timed pulses of Shh signalling on neural progenitor proportions. First, we simulated the effect of 24 h with no SAG followed by 48 h with 500 nM SAG. Under the hypothesis of instantaneous landscape responses, the increase in SAG would trigger immediate bifurcations, destabilising dorsal states (p0-p2). This predicted that, at D4, after 24 h with 0 nM SAG, cells would adopt a p0/p1 state, at which point an abrupt increase to 500 nM SAG would destabilise this state, driving the cells to transition further into the intermediate sub-landscape and not into the usual ventral route to p3. Since the p2 state is absent at 500 nM SAG, cells would rapidly adopt a pMN* identity. Importantly, because most cells are predicted to have left the PreNeural attractor and committed to an intermediate fate by D4, few PreNeural cells would be left to transition to the ventral sub-landscape. Consequently, under this hypothesis, the model predicts substantially fewer p3* cells and a purer population of pMN* at D5-D6 compared to constant 500 nM SAG exposure.

We experimentally tested this hypothesis by culturing cells in the absence of SAG for 24 h, from D3 to D4, and then adding 500 nM SAG for the subsequent 48 h (D4-D6). We analysed the proportions of cell types at D4, D5, and D6 using flow cytometry. The experimental data were in good agreement with the simulations, with the majority of cells adopting a pMN* identity at D6 (Fig 12D). These results indicated that the response to raising levels of SAG resulted in fast predictable changes to the landscape.

We next simulated the opposite pulsing regime—500 nM SAG for 24 h followed by 0 nM SAG for 48 h. to explore the impact on the landscape of an abrupt removal of SAG at D4. Assuming cells respond immediately to the removal of the signal, triggering instantaneous bifurcations in the landscape, the model predicts the reestablishment of the p0/p1 state and cells that had not reached the p2 state by D4 would remain trapped in p0/p1. Consequently, we would expect a higher proportion of p0/p1 cells to persist at D6 compared to continuous exposure to 500 nM SAG.

Experimental results showed a markedly different outcome from this prediction: pMN* cells dominated at D5 and D6 and p0/p1 cells were largely absent as in the constant exposure case (Fig 12E). The almost complete absence of p0/p1 indicated an irreversible commitment to pMN* fates and that the cells did not respond to an instantaneous signal withdrawal. This is consistent with previous experimental observations of bistability and hysteresis in neural progenitor specification [72].

The similarity in cell states proportions between constant 500 nM exposure and SAG removal after 24 h rules out the assumption of an instantaneous cellular response to signal withdrawal. To distinguish whether the response to withdrawing SAG is delayed or entirely absent, we fitted the model directly to the experimental data (Fig 12E) and compared the resulting PPD ‘post-500 nM-SAG’ with that from continuous 500 nM SAG exposure. The PPDs were identical (Fig 12B), indicating that the landscape remains unchanged after SAG withdrawal and the dorsal states (p0-p2) are not reestablished. Similar results from 100 to 0 nM SAG transitions demonstrate that 100 nM SAG is also sufficient for irreversible p0/p1 destabilisation (Fig 12F). Further analysis, including an experiment using a shorter 12 h pulse of 500 nM SAG, confirmed that the sustained landscape effect persists throughout the post-withdrawal period (Section B6 in S2 Appendix).

**Experimental validation of convergent topology.** Finally, the global model predicts that, at 500 nM SAG, a substantial proportion of cells reach p3 via the intermediate DP route, arriving later than cells taking the direct ventral pathway. Consistent with this prediction, scRNA-seq data showed concurrent DP depletion and p3 accumulation between D6 and D7 (Fig 11B).

**Fig 13:**
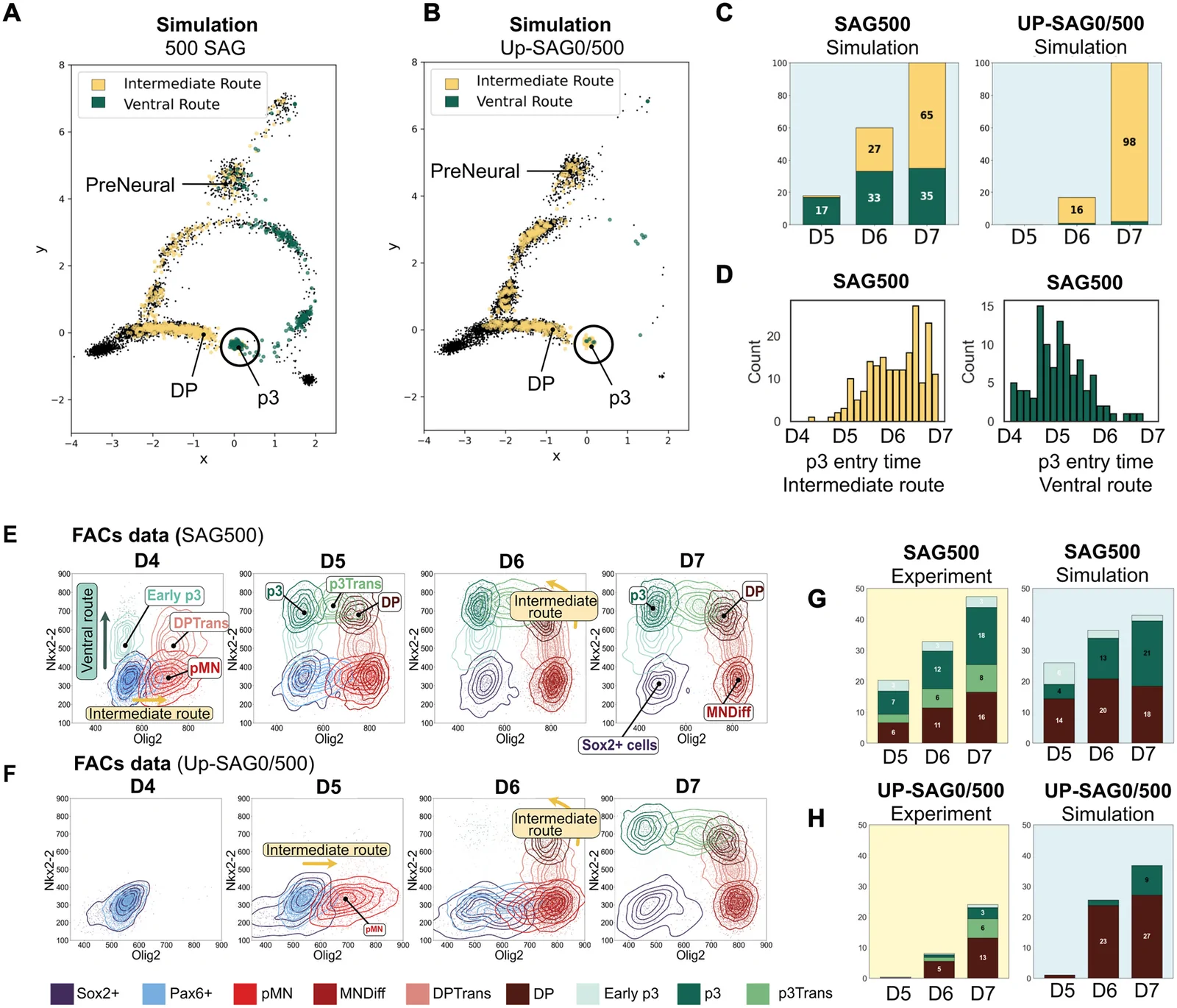
Validation of the DP-to-p3 transition. **A.** Simulated cell trajectories using the PPD from model fitting (500 nM SAG condition). Dots represent simulated cells retained at 4 time points (D4-D7). Cells reaching the p3 AC by D7 are coloured based on their transition history: yellow for cells that follow the intermediate route and green for those that follow the ventral route. Black dots represent cells that adopted alternative end fates (such as MNDiff or FP) or have not yet reached p3 by D7. **B.** Similar to A, but simulating a delayed pulse of SAG by switching the parameters from 0 to 500 nM SAG after D4. Substantially fewer cells follow the ventral route as most cells had already taken the intermediate route by D4 when the signal changed. **C.** Proportions of simulated cells in the p3 AC for each day (D5 to D7) computed with respect to the total number of p3 cells at D7 and coloured by the route they originated from. (Left) 500 nM SAG. (Right) Up-SAG0/500 nM SAG condition. The majority of p3 cells under this condition originate from the intermediate route. **D.** Histograms showing cell counts (y-axis) by the time point (x-axis) at which simulated cells first reach the p3 AC, with colours indicating their route of origin: intermediate (yellow) or ventral route (green). The distributions reflect timing heterogeneity: cells that reach p3 via the intermediate route tend to arrive at late time points (D6-D7). By contrast, cells following the ventral route tend to reach p3 early (D4-D5) while few new cells arrive via this route after D6. **E, F.** 2D Gene plots (Olig2-Nkx2.2 shown) of the clusters identified in the flow cytometry dataset at each time point. The dots represent individual cells, and contour lines represent the density of cells (plotted only when the cluster contains more than 150 cells). Without Nkx6.1 p0/p1 cannot be distinguished from p2 cells and both are combined into Pax6+ cells. PreNeural, EarlyVentral and FP are combined into Sox2+ cells. E. 500 nM SAG: as early as D4, a group of cells follow the ventral route by up-regulating Nkx2.2 and reach p3 by D5. F. Up-SAG0/500 nM SAG condition: with no SAG for 24 h, cells are forced to take the intermediate route and most of them had up-regulated Pax6 by D4. When SAG is applied, they can only reach p3 by transiting through pMN (D5), then DP (D6), followed by a direct DP-to-p3 transition (D7). **G.** (Left) 500 nM SAG: experimental proportions of the DP AC and p3Trans (intermediate route) and the Early p3 and p3 ACs (ventral route) at late time points (D5-D7). (Right) 500 nM SAG: simulated proportions of the DP, Early p3 and p3 ACs at late time points (D5-D7). The p3 proportions can be further split into two type of cells depending on which route they followed. **H.** Same as G for the Up-SAG0/500 nM condition. In the simulation (right), most p3 cells at D7 originate from the intermediate route as shown in B which is consistent with the experiment F. The data underlying this Figure can be found at https://crick.figshare.com/projects/Neural_Tube_Decision_Landscape/250460 and at https://doi.org/10.5281/zenodo.15584010.

To test this prediction, we simulated two signalling regimes: constant 500 nM SAG versus 24 h at 0 nM followed by 72 h at 500 nM SAG (Fig 13A, 13B). We tracked which route cells followed to reach p3. Under the second signalling regime, the vast majority of p3 cells took the intermediate DP-to-p3 route rather than the ventral route via EarlyVentral and Early p3 (Fig 13C). Entry time analysis revealed distinct kinetics for the two pathways. Cells reached p3 earlier via the ventral route, whereas the intermediate route resulted in delayed arrival due to retention in shallow intermediate states (Fig 13D).

To test these predictions, we performed experiments extending the time course to D7 (Fig 13E, 13F) and analysed the effect of adding 500 nM SAG at D4 after 24 h without SAG. Under these conditions, the model predicts that PreNeural cells are forced to take the intermediate route as almost all cells initially transition to p0/p1. Subsequently, elevating SAG levels to 500 nM SAG destabilises the p0/p1 and p2 attractors, making p3 accessible via the DP saddle. Flow cytometry data at D7 revealed that cells had transitioned from DP to p3, characterised by decreased Olig2 expression while maintaining Nkx2.2 (Fig 13F). This is particularly clear at D7. In conditions in which SAG exposure is delayed by 24 h, p3 cells emerge despite the near absence of Early p3 at earlier time points, ruling out the possibility of a ventral route.

We compared the simulated proportions of DP, Early p3 and p3 ACs for both experimental conditions (Fig 13G, 13H), using the PPDs previously obtained for 0 and 500 nM (this experiment was not included in the fitting of the model). The model captured the increased proportion of DP cells and the passage from DP to p3 via the intermediate route (Fig 13H) (S3–S4 Videos).

Together, these results further validate the model and demonstrate the complex relationship between signal dynamics and cell fate allocation. The analysis indicates that transient high-level Shh signalling induces irreversible commitment to ventral fates, while also providing support for the dual pathways to p3 identity. These findings establish how cells integrate and retain signalling information to make robust fate decisions, with the mathematical model providing a framework for understanding the underlying dynamical principles.

### Testing DLA on *in vitro* human data

To evaluate the generality of the landscape model, we investigated its applicability to an in vitro model of human trunk development [38]. This system generates multiple embryonic tissues, including neural progenitors, mesodermal cells, and notochord cells, which are a source of endogenous Shh signalling to the other tissues. The presence of the Shh producing notochord in these organoids results in the generation of ventral neural tube progenitors (p2, pMN, p3) and floor plate (FP). We analysed two experimental conditions - an *18h-delay* and *24h-delay* in TGFβ addition - which produced different proportions of notochord cells and consequently varying levels of Shh signalling [38]. The data comprised scRNA-seq collected at 3 time points D3, D5, and D7.

**Fig 14:**
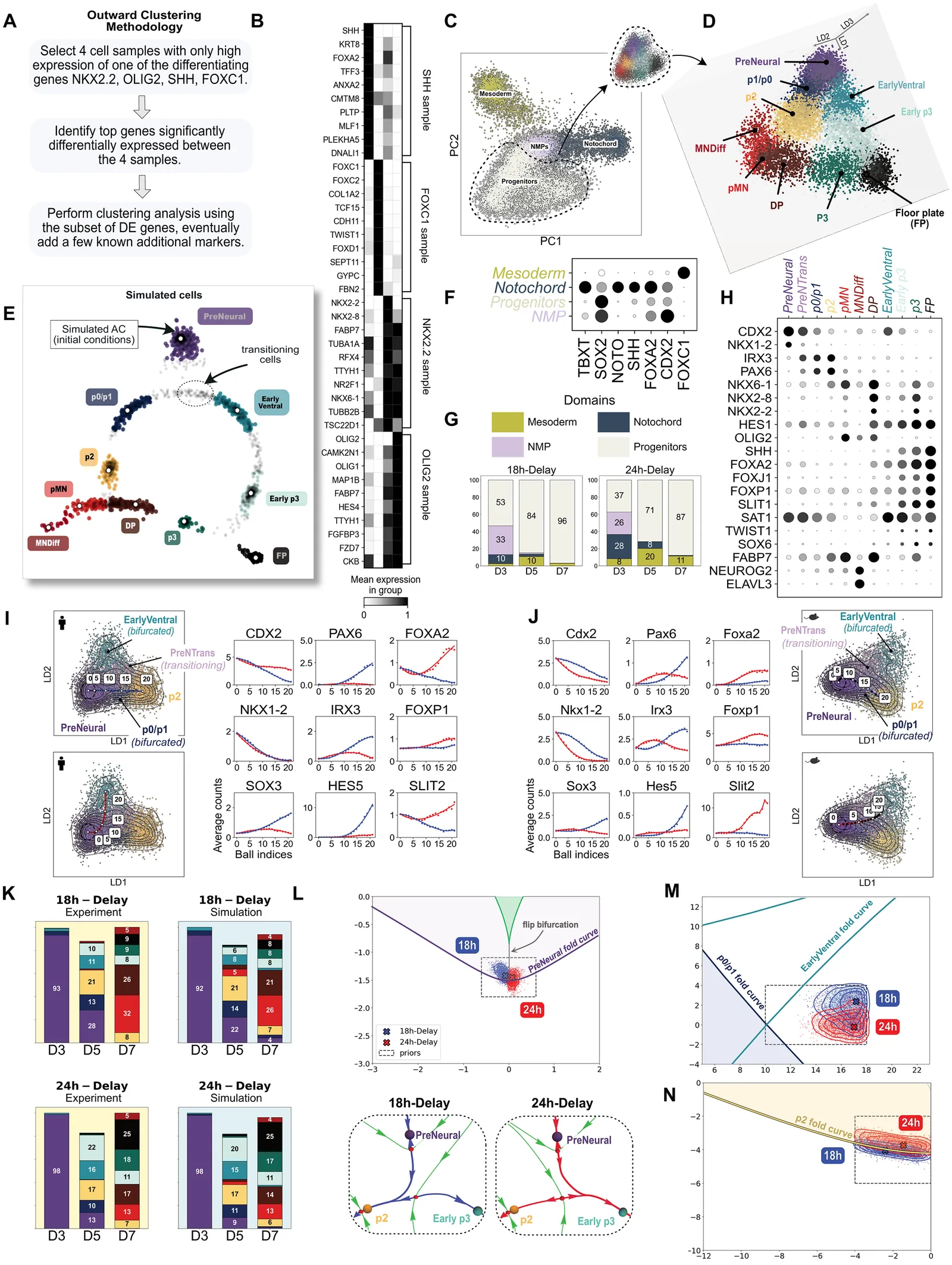
Analysis of human neural progenitor development validates the circular topology of the decision landscape. **A.** Summary of the outward clustering methodology for the human organoid data. **B.** The most significantly differentially expressed genes found using the 4 samples defined in A. **C.** The four cell type domains including neural progenitors were extracted, annotated and separately projected using LDA from the effective gene space defined by the gene modules in B. **D.** 3D LDA projection of the neural progenitor ACs for all time points and experimental conditions. This reveals the circular topology of the transition pathways. **E.** Simulated neural progenitor ACs produced by the landscape model of this system after fitting in a similar fashion to that described above. Dots represent simulated cells retained at 3 time points corresponding to D3, D5, D7 using the parameters from the fitted PPDs for the 18h-Delay condition. Grey dots represent transitioning cells that were not assigned to any simulated AC. **F.** Dot plot indicating the expression of markers used to identify the tissue domains. The intensity of shading indicates the mean expression of each gene in each domain. **G.** Proportions of cells in each domain of C and F by time point in the two experimental conditions. **H.** Dot plot indicating the expression of markers used to identify the ACs and transitioning clusters of the Neural Progenitors domain in D. **I, J.** The PreNeural sub-landscape extracted from the human dataset (I) and the mouse datasets (J) and transition analysis for marker genes that characterise the binary decision as cells exit PreNeural. Unstable manifolds defining routes between pairs of ACs were computed in effective gene space defined by the genes in B and projected onto 2D LDA space. Marker genes were analysed along both routes. Exit from PreNeural is marked by down-regulation of Cdx2 and Nkx1.2. A branching event occurs (around index 10), followed by up-regulation of markers characteristic of ventral states along the red route (leading to EarlyVentral), and markers such as Pax6 characteristic of the intermediate states p0/p1, and p2 along the blue route. **K.** (Top) Comparison of the experimental and simulated proportions after parameter fitting for the 18 h-Delay condition. (Bottom) Similar for the 24 h-Delay condition. This shows an increase of ventral states such as p3 and FP. **L.** (Top) Bifurcation set of the PreNeural sub-landscape shows the qualitative difference between the 18 h- and the 24 h-Delay conditions. The 18h-Delay PPD (blue) appears on the left of the flip curve and above the PreNeural fold curve. Under this condition, the PreNeural attractor is shallow for most cells and is connected to an intermediate fate. By contrast, the 24 h-Delay PPD (red) lies to the right of the flip curve, favouring cell trajectories from PreNeural to a ventral fate and promoting the generation of more p3 and FP cells. (Bottom) Typical configurations of attractors, saddles and unstable manifolds for the cell landscape models for the PreNeural sub-landscape for parameters in each of the PPDs. In the 18 h-Delay, cells are most likely to transition via the intermediate route towards p2 and to Early p3 via the ventral route in the 24 h-Delay. As indicated by the PPDs in M, the attractors p0/p1 and EarlyVentral are bifurcated for all cells in both experimental conditions. Hence, the connections from PreNeural lead directly to p2 (blue unstable manifold) or to Early p3 (red unstable manifold). **M.** Bifurcation set of the sub-landscape controlling the p0/p1 and EarlyVentral fold bifurcations. Under both conditions, the PPDs are in the region where the p0/p1 and the EarlyVentral attractors are bifurcated for all cells. **N.** Bifurcation set of the p2 transition shows that, under both conditions, the p2 AC is shallow as most parameters cluster near (and above) the p2 fold curve in the shaded region. The data underlying this Figure can be found at https://doi.org/10.5281/zenodo.15584010.

Using the clustering method described above (Fig 14A, 14B), we identified the expected cell populations: NMPs, mesoderm, ventral neural progenitors, and notochord (Fig 14C, 14F, 14G). When examining neural progenitors (SOX2^+^, TBXT^-^), we discovered remarkably similar ACs to the mouse dataset. Moreover, an LDA projection revealed the ACs were arranged in a similar pattern to the previous data: the PreNeural state leading to intermediate and ventral sub-landscapes and comparable transitions between ACs including a circular topology in which the two routes converge to the p3 AC (Fig 14D – 14E).

#### Conservation of the decision landscape topology.

Despite differences in developmental timing, gene expression patterns characterising the ACs (Fig 14H) and individual analysis of each sub-landscape showed striking conservation between mouse and human systems (Fig 14I, 14J for the PreNeural sub-landscape and Figs A16-A17 in S1 Appendix for all the other sub-landscapes). We therefore fitted our landscape model to the two datasets *18h-delay* and *24h-delay* focussing on the neural progenitor landscape. We anticipated that, since the *18h-delay* condition contains less notochord and therefore less Shh signalling than the *24h-delay* condition, the *18h-delay* dataset would behave as if it had been exposed to lower SAG concentrations than the *24h-delay* dataset. Cells in the D3 PreNeural AC were used as initial conditions and comparisons were made to the proportions of cells in each simulated AC with the experimental cell states proportions. The model successfully reproduced the experimental data (Fig 14K). Details of the fitting and the simulation can be found Section B9 in S2 Appendix.

#### Using bifurcations to quantify the effect of notochord-derived Shh on the decision landscape.

Analysis of the bifurcation sets revealed key differences between the *18h-delay* and *24h-delay* conditions. The 24h-delay generated more notochordal cells (Fig 14G), resulting in higher proportions of floor plate and p3 progenitors and fewer pMN cells by D7 than the 18h-delay. Importantly, the bifurcation analysis showed that these differences primarily affected the PreNeural flip bifurcation (Fig 14L), while other bifurcations remained unchanged between conditions (Fig 14M, 14N). In both experimental conditions, the p0/p1 attractors were unstable across all cell landscapes, making the p0/p1 AC transient - mirroring the behaviour observed at high SAG concentrations in the mouse experiments (Fig 14M). Most p2 attractors remained shallow, with the corresponding PPDs clustering just within the p2 bifurcation fold curve (Fig 14N). This suggests that the increased number of notochordal cells in the 24-delay condition, and consequently the higher level of Shh signalling, primarily influenced the first neural progenitor cell fate decision, directing more cells towards the ventral sub-landscape, providing quantitative insight into how notochord-derived Shh signalling affects ventral patterning.

These findings demonstrate that the landscape model successfully captures neural patterning dynamics across species while revealing features of human development. The analysis identified how cell state transitions are affected by notochord-derived Shh: while the PreNeural flip bifurcation showed high sensitivity, other transitions displayed qualitative robustness to signalling variations. This raises important questions about how dorsal fates are established in the presence of strong ventralising signals from the notochord, suggesting the necessity of additional regulatory mechanisms. Future studies will need to explore how combinations of signals shape the early neural tube landscape.

## Discussion

### Dynamic Landscape Analysis: framework and methodology

In this study, we developed and applied DLA, a systematic framework for constructing quantitative and predictive landscape models from single-cell data. By applying this approach to a model of neural tube development, we uncovered an extensive landscape with a rich bifurcation structure and an unexpected circular topology, where initially divergent lineages converge through multiple routes.

A key component of this approach was the identification and characterisation of ACs. This follows a semi-supervised strategy, starting with known marker genes and iterative expansion to identify genes associated with the ACs and the transitions between them. This approach identified established neural progenitor states and less well-understood states, such as a double-positive population expressing both Olig2 and Nkx2-9, along with Hes1 [58]. The method is characterised by greater control of dimensionality, motivated by the need to avoid noisy or irrelevant dimensions in the data and the extensive experimental work suggesting that cell fate decisions are controlled by a relatively small number of genes [1]. While current techniques for analysing scRNA-seq data often start with highly variable genes (typically numbering in the thousands), the ratio of relevant genes to all features in such samples is small. As the fraction of relevant features decreases, existing clustering and dimensionality reduction techniques often fail to discover the identity of relevant features because correlations between samples become dominated by noise and fluctuating irrelevant genes [73]. Moreover, since understanding transition mechanisms and branching points is a key priority, optimal observability of transition routes is crucial and this requires the ability to compute cell fate differentiation paths in the gene expression space as opposed to tracing them on a 2D or 3D visualisation. The relatively low dimensionality of our gene modules facilitates this.

A second important feature of DLA is the use of dimension reduction methods that preserve the geometry of the local landscape and the transitions. Our analysis of the bifurcation structures depends upon the dimension reduction projection being differentiable. Noncontinuous or nondifferentiable methods would eliminate critical geometric features, particularly unstable manifold smoothness. While neural networks can be used to modify t-SNE [74] and UMAP [75] to create smooth embeddings [76,77], LDA provides superior results for analysing related attractors because it maximises separation through a controlled, understandable linear embedding.

Finally, DLA was designed so that it can handle multiple datasets across different time points and morphogen levels without data integration, enable quantitative comparisons between such datasets, and accommodate multiple data modalities, including scRNA-seq and flow cytometry.

### Attractor dynamics, shallow attractors, and the limits of pseudo-time

The availability of a fitted stochastic model that reproduces experimental data allows a better understanding of analytical methods for single-cell data. For example, there has been much interest in trajectory inference methods which aim to infer a 1-dimensional graph-like structure underlying the dynamic process from which the cells are sampled [78,79]. By projecting the cell states to this structure, their properties can be compared over pseudo-time, an inferred unit of progress along the graph-like structure. In this approach, the cell state branches correspond to nodes of the graph with one inward edge and two outward edges. Flip bifurcations have a similar branching topology, but cell states traverse it with radically different dynamics to that given by pseudo-time.

Our theory predicts that at the branching point there is a head attractor that cells pass through. Experimental results and models validate this. Cells can either remain in a strong attractor until a signal triggers a bifurcation, or linger near a shallow attractor for an extended period before escaping through stochastic fluctuations (Fig 10B). Consequently, a global pseudo-time cannot accurately represent a cell’s passage through such an attractor. Simulations demonstrate this limitation, showing cells arriving at p3 via the intermediate route are approximately equally distributed across days D5, D6, D7, despite starting from the NMP state simultaneously (Fig 13D). By contrast, pseudo-time aligns more closely with actual simulation time during transitions between attractors (Fig 10B). The “stickiness" of attractors explains the difficulty in capturing transitioning cells in experimental data with limited temporal resolution.

### Shallow attractors, ghost fixed points, and the limits of snapshot data

An important conceptual question is whether head attractors from which leakage is observed represent genuine shallow attractors, or *ghosts* characterised by regions where cells transition slowly near an already bifurcated attractor [80,81]. Our approach addresses this ambiguity operationally through analysis across a range of times and experimental conditions. A true AC should persist across relevant conditions and bifurcate in a characteristic way when some change. Moreover, the fitting of the landscape model provides information about this by tracking whether the PPD lies within the region bounded by the fold curve (Fig 11F). Finally, the analysis associated with Fig 5F can address the difference between a ghost and a true, but shallow, attractor from which cells leak at a roughly constant rate. In the former case we expect to observe a pulse of cells exiting an AC and proceeding down the transition route seemingly in unison as in Fig 5F, while in the latter case the distribution shown in this figure will not progress but remain stationary.

Fully resolving this requires perturbation experiments aiming at stabilising the head attractor to reduce the observed leakage from it. The p2 state (also p0/p1) provides a clear example: it functions as a stable state at 10 nM SAG but appears as a transitioning state at 500 nM SAG (Fig 6H). We note that this ambiguity does not affect the predictive utility of the framework, since the same landscape model describes both regimes: in the example of the p2 state, the PPD of the fitted model lies within the region bounded by the fold curve (Fig 11F) at 10 nM SAG but below it at 500 nM SAG. We note that the theoretical framework accommodates both interpretations equally, and the ghost attractor scenario has been proposed as a timing mechanism in other developmental contexts [82].

Our data consist of population snapshots throughout, which places fundamental limits on inferences about individual cell behaviour. We cannot, for example, exclude the possibility that cells switch between trajectories, especially soon after exiting a head attractor. It is also possible that there are transition shortcuts in dimensions not captured by our gene module space. However, this is less likely as we would pick up any of those extra genes that are differentially expressed between the ACs. Moreover, if the cells in this route were plentiful this would affect the proportions occupying each of the relevant cell states making it hard for the model to consistently fit the data. We consider the interpretation we provide the most parsimonious consistent with the data, supported by the coherent structure of transitioning cell distributions and quantitative agreement between the model and the experiments.

On the other hand, the availability of snapshots at multiple time points and under different experimental conditions allows not only the qualitative understanding of the landscape but also how it changes under signalling variation. This and the quantitative single-cell data allows the development of a stochastic mathematical model that quantitatively and efficiently summarises all this information as well as enabling the prediction of unseen scenarios.

### Biological implications of bifurcation types

The bifurcation types provide insight into biological features of cell fate decisions. Fold bifurcations explain how an incremental change in signal strength can trigger a switch in cell identity once a threshold value is crossed. On the other hand, a binary flip bifurcation describes the concurrent production of two downstream states from a progenitor population. In this case, differences in cell state or signals received by a cell determine which of the two possible escape routes a cell chooses. Thus, at a population level, two fates can be generated simultaneously. The flip bifurcation implies that a cell commits to differentiate from the head attractor before specifying which fate it will choose. This architecture could explain the phenomenon of multi-lineage priming, where cells simultaneously express low levels of genes associated with multiple potential fates before committing to a specific lineage [63].

### Convergent lineages and future experimental tests

A striking feature of our analysis is the circular topology of the neural progenitor landscape, where two developmental trajectories converge on the p3 identity. Experimental perturbations confirmed that p3 cells can be generated through both paths, with the route followed depending on the timing and level of Shh signalling. Applying our landscape model to human neural organoid development revealed consistency between human and mouse systems, suggesting that this circular topology represents a fundamental feature of vertebrate neural development. This finding reconciles previously contradictory findings of the p3 domain being generated through either a Foxa2^+^/Pax6^−^ route [36] or through a Pax6^+^/Olig2^+^ route [64]. It also suggests greater complexity and redundancy in developmental programmes than can be explained by the conventional view of a strictly hierarchical ‘tree’ of cell type diversification. In development, distinct lineages derived from the same progenitor pool can later reconverge; haematopoiesis is one example [83].

The transition routes inferred by DLA make specific predictions that are testable with clonal lineage analysis. The circular topology in which p3 cells can be generated via two distinct routes predicts that cells marked at the PreNeural stage should produce p3 progeny with distinct kinetics depending on which route is followed. Retrospective lineage tracing (e.g., barcoding or single-cell clonal assays in the in vitro differentiation system [84,85]) would allow the ancestry of p3 cells to be traced to either Foxa2^+^/Pax6^−^ precursors (ventral route) or Pax6^+^/Olig2^+^ precursors (intermediate route). Similarly, binary flip architectures predict that sister cells within a clone can adopt divergent fates, whereas direct transitions predict near-homogeneous clonal outcomes. This in vitro system is particularly amenable to such approaches and we regard lineage tracing combined with DLA as the natural next experimental step.

### Implications for GRN modelling and predictive parameter inference

While GRN models have been successfully constructed for some systems, there has been a notable lack of progress in others, and current single-cell computational approaches have been disappointing [86]. GRN construction has been feasible in systems such as oscillators where there is an obvious dynamical phenotype that constrains the possible dynamics of the GRN with easy and informative readouts such as period, amplitude and phase that can be used to explore the effects of perturbations. The generative modelling approach we have developed could play an analogous role for other systems by providing similar tools such as a clear phenotype (the route and timing of a cell passing through the ACs and the proportion of cells doing this) and a precise prescription of the dynamical phenomena that the GRN model must display. Moreover, the simplest gene circuits that produce the local normal forms will likely be highly constrained and this will provide a limited set of gene interaction modules that can be searched for in the data [17,19].

Our approach to modelling allows us to readily identify the bifurcation set in the model parameter space. Combining this with the PPD found by parameter optimisation provides a potentially powerful tool. It links the PPDs corresponding to different morphogen levels to dynamical behaviour. The relationship between the estimated PPD and the bifurcation curves predicts the behaviour of a heterogeneous group of cells exposed to a particular level of signal. For example, in cell states with stable identities, such as p0/p1 in low SAG concentrations, we find parameters clustering away from critical bifurcation curves so that even a heterogeneous cell population displays homogeneous behaviour. In other cases, the PPD overlaps bifurcation curves in a way that suggests a cell population makes a mixture of alternative decisions (e.g., to transition, remain in place, or choose between options in a binary flip landscape). For different morphogen levels all are seen in the transition from the PreNeural state (Fig 11G). More varied cellular outcomes can occur when the PPDs for different morphogen levels overlap more complex structures in the bifurcation curves, as is the case for the transition from pMN to MNDiff or DP (Fig B8 A Panel 5 in S2 Appendix). A key insight is that distinct fate choices for a group of initially near-identical or "microheterogeneous" cells [87] can emerge from the deterministic landscape dynamics (bifurcations) rather than solely from noise induced stochastic fluctuations affecting the trajectory of a cell [88].

By systematically characterising the decision points and the parameters that control cell fate decisions, we can design differentiation protocols that precisely manipulate bifurcation parameters towards desired cell type proportions. This systematic approach provides a deeper understanding of cellular decision-making providing a precise and quantitative approach for designing in vitro protocols which, in time, could be applied to organoid models, stem cell therapies and regenerative medicine.

### Hierarchical decision structure, spatial patterning, and robustness

An unexpected insight arising from the analysis of the model is that it challenges the conventional hypothesis for morphogen patterning of a tissue in which monotonically ordered morphogen thresholds directly determine domain boundaries. Instead, the analysis suggests that cells progress through a series of binary decisions, first choosing between the intermediate or ventral route and then patterning according to monotonic thresholds or further flips within the separate routes. Critically, the flip bifurcations at PreNeural and Early p3 attractors reveal that the pMN/p3 and p3/FP boundaries in the embryo arise from branching decisions rather than direct transitions between adjacent states. This involvement of flip bifurcations in establishing developmental boundaries introduces a hierarchical component to spatial patterning and expands the repertoire of morphogen-dependent pattern-forming mechanisms. It is consistent with recent evidence of distinct epigenetic regulatory states distinguishing p3 and pMN [36]. We conjecture that this hierarchical decision-making architecture, coupled with the circular landscape topology, provides greater dynamical robustness compared to simple sequential monotonic thresholds.

Intriguingly, similar hierarchical decision-making dynamics have been observed in the learning process of generalised Hopfield networks, where memories progress through well-defined saddles before splitting into progressively specialised states [89]. In both neural development and machine learning, these decision landscapes appear to follow reproducible, low-dimensional trajectories despite the high-dimensional nature of the underlying systems. This similarity between biological cell decision-making and machine learning suggests that hierarchical binary decisions may represent a fundamental organising principle for robust decision-making across different complex systems.

### Future directions

A challenge for future work is to extend this DLA framework to describe how combinations of different signals shape developmental decision-making by including a model of the way such multidimensional signals change the parameters of the model, as was done for a simpler system [18]. For these more sophisticated applications, integration of epigenetic data with transcriptional profiles may be required.

Taken together, DLA provides a framework for generating predictive mathematical models of cell fate decisions from single-cell data. With this framework, we have gained insight into how cells navigate fate decisions in neural development. By bridging the gap between high-dimensional single-cell data and mathematical models of cellular behaviour, DLA can advance our understanding of the principles governing cellular differentiation.

## Materials and methods

### Cell lines

Experiments were performed with the mouse embryonic stem cell line HM1 [90] maintained at 37 °C with 5% CO_2_.

### ES cell culture and differentiation

Mouse ES cells were maintained on a feeder layer of mitotically inactivated mouse embryonic fibroblasts (MEFs, derived and expanded in-house) in ES cell medium (Dulbecco, Modified Eagle Medium (DMEM) Knock Out (Gibco; 765 10829-018) supplemented with 1% Foetal Bovine Serum (Pan Biotech; P30-2602), Penicillin/Streptomycin (Gibco; 766 15140122), 2 mM L-Glutamine (Gibco; 25030024), 2 mM Nonessential amino acids (Gibco, Cat No. 11140-035), and 0.1 mM 2-mercaptoethanol (Gibco; 21985-023)) with 1000 U/ml LIF (Chemicon, Int ESG1107)). Media was 768 changed every day and cells were passaged every other day at a density of 500,000 cells per 60mm dish.

For differentiations, cells were washed once with PBS and dissociated using 0.05% Trypsin-EDTA (Gibco; 25300054) for 4 min at 37 °C and resuspended in 10 ml ES media. Cells were plated in 10 cm plates coated with 0.1% gelatine to remove feeder cells (‘panning’). Cells were incubated for 15 min at 37 °C to allow feeder cells to attach. Without disturbing any attached cells, the cells in suspension were transferred to a second gelatineised 10 cm for another 15 min. The process was repeated a third time.

Differentiations were carried out for this study using previously published differentiation protocols [36], by plating ‘panned’ mES cells resuspended in N2B27 media (Advanced DMEM - F12 (Gibco, Cat. No. 21331-020) and Neurobasal medium (Gibco, Cat. No. A35829-01) (1:1), supplemented with 1xN2 (Gibco Cat no. 17502001), 1xB27 (Gibco Cat no. 17504001), 2 mM L-glutamine (Gibco, Cat No. 25030024), 40 μg/ml BSA (Sigma-Aldrich, Cat No. A7979-50ML), and 0.1 mM 2-mercaptoethanol) at a density of 20,000 cells in 1.5 ml of media onto 6-well plates (Corning, Cat. No. 353046) precoated in Matrigel (Corning, Cat. No. 356231) diluted 1/100 in Advanced DMEM - F12. The media was supplemented on the different days as follows: Day 0 to Day 2, 10ng/ml bFGF (R&D, Cat. No. 100-18B) and 5 μM LGK (Cayman Chemical Company, Cat. No. 1.800.364.9897); Day 2 to Day 3 for 20 h, 10 ng/ml bFGF, 5 μM CHIR99021 (Axon Medchem, Cat. No. 1386), 10 μM SB-431542 (Tocris, Cat. No. S0400), and 2 μM DMH1 (Adooq Bioscience, Cat. No. A12820); from Day 3 onwards, 100 nM RA (Sigma, Cat. No. R2625) and the indicated concentrations and timings of Smoothened Agonist SAG (Merck, Cat. No. 566660-5mg).

### Intracellular flow cytometry

Sample collection: 1 μl/ml of LIVE/DEAD Fixable Dead Cell Stain Near-IR fluorescent reactive dye (ThermoScientific, Cat. No. L34976) was added to cells in culture and incubated at 37 °C for 30 mins. Cells were then washed twice with PBS (Gibco, Cat. No. 14190-094), and dissociated using 0.5 ml Accutase (Gibco, Cat. No. 00-4555-56) per well of a 6 well plate incubated 5 min at 37 °C. Cell were collected, centrifuged at 400 *g* for 4 min and resuspended in 100 μl of 4% paraformaldehyde (PFA) (ThermoScientific, Cat. No. 28908). PFA fixation was carried out for 10 min at room temperature. Cells were washed in PBS and resuspended in 500 μl PBS + 0.5% BSA.

Staining: 1 million cells were stained for flow cytometry analysis. Pellets were resuspended in 0.1 ml of PBS supplemented with 0.5% BSA and 0.1% Triton-X100 (VWR Chemicals, Cat No. 28817.295) and the appropriate primary or directly-conjugated antibodies for 1.5 h protected from light at room temperature. Secondary antibodies were incubated under the same conditions for 40 min. Cells were washed in PBS supplemented with 0.5% BSA and 0.1% Triton-X100, and resuspended in 300 μl of PBS with 0.5% BSA for analysis on a BD Fortessa analyser (Becton Dickinson).

The antibody panels used were as follows: Primary antibodies were Sox2-V450 (1:100)(BD Biosciences Cat no 561610), Pax6-PerCPCy5.5 (1:100) (BD Biosciences Cat no 562388), Nkx6.1-AlexaFluor647 (1:100)(BD Biosciences Cat no 563338), Goat Olig2 unconjugated (1:400) (R&D Cat no AF2418) and Nkx2.2-PE (1:100) (BD Biosciences Cat no 564730) followed by secondaries donkey anti-goat AlexaFluor488 (1:1000) (Thermo Fisher Scientific cat no A11055).

### Flow cytometry data pre-processing

The flow cytometry data were analysed using FlowJo v10.8 Software (BD Life Sciences). Cells were selected for downstream analysis if they were negative for LIVE/DEAD Fixable Dead Cell Stain Near-IR (alive), and with uniform FSC, SSC distribution. The cells were also required to be SOX2+ (neural progenitors).

## Supporting information

S1 Appendix Detailed description of the data analysis. (PDF)

S2 Appendix Detailed description of the modelling. (PDF)

S1 Video Simulation 10 nM SAG. (MP4)

S2 Video Simulation 100 nM SAG. (MP4)

S3 Video Simulation 500 nM SAG. (MP4)

S4 Video Simulation Up–SAG0/500. (MP4)

S1 Table Summary of the flow cytometry experimental datasets, included as an attached file. (DOCX)

## Abbreviations

ACs: attractor clusters
DLA: dynamic landscape analysis
DMEM: Dulbecco, Modified Eagle Medium
EPSRC: Engineering and Physical Science Research Council
FP: floor plate
GRNs: gene regulatory networks
KITP: Kavli Institute for Theoretical Physics
LDA: linear discriminant analysis
MEFs: mouse embryonic fibroblasts
NMPs: neuromesodermal progenitors
NMPTrans: transitioning NMPs
PCA: principal component analysis
PFA: paraformaldehyde
PPD: posterior parameter distribution
Shh: Sonic Hedgehog.
