# Supplementary material for: Dynamic Landscape Analysis of cell fate decisions provides predictive models of neural development from single-cell data
Source: PLoS Biol. 2026 Aug 26;24(8):e3003953. doi: 10.1371/journal.pbio.3003953 (PMC13588503; doi:10.1371/journal.pbio.3003953)
Supplement: Supplementary file 3 [file pbio.3003953.s007.docx]

| **Signaling Regime** | **Timepoints** | **ReplicateDate** | **doi** |
| --- | --- | --- | --- |
| SAG switch after 24h (0 nM to 500 nM) | Days 4-7 | DiffCom08July | https://doi.org/10.25418/crick.29155301.v1 |
| SAG switch after 24h (0 nM to 500 nM, 500 nM to 0 nM) | Days 3-6 | DiffCom22Oct, DiffCom5Nov | https://doi.org/10.25418/crick.29155280.v1 |
| SAG pulse (0 nM for 0-12h, 500 nM 12-24h, 0 nM SAG onwards) | Days 4-6 | DiffComJune2024 | https://doi.org/10.25418/crick.29155286.v1 |
| SAG switch after 24h (100 nM to 0 nM) | Days 4-6 | DiffComJune2024 | https://doi.org/10.25418/crick.29155289.v1 |
| Constant SAG (0 nM, 10 nM, 100 nM, 500 nM) | Days 4-6 | DiffCom16Oct | https://doi.org/10.25418/crick.29155244.v1 |
| Constant SAG (0 nM, 10 nM, 100 nM, 500 nM) | Day 4, Day 6 | DiffCom30Oct | https://doi.org/10.25418/crick.29155268.v1 |
| Constant SAG (0 nM, 10 nM, 100 nM, 500 nM) | Days 3-6 | DiffCom12March | https://doi.org/10.25418/crick.29155223.v1 |
| Constant SAG (0 nM, 10 nM, 100 nM, 500 nM) | Days 3-6 | DiffCom16Apr | https://doi.org/10.25418/crick.29155241.v1 |
