# Supplementary material for: Dynamic Landscape Analysis of cell fate decisions provides predictive models of neural development from single-cell data
Source: PLoS Biol. 2026 Aug 26;24(8):e3003953. doi: 10.1371/journal.pbio.3003953 (PMC13588503; doi:10.1371/journal.pbio.3003953)
Supplement: Supplementary file 4 [file pbio.3003953.s008.docx]

Response to reviewers comments

We are very grateful to the reviewers for the effort they have put into understanding our paper. We believe their suggestions have helped us significantly improve the exposition. In this response, we are providing a PDF version of the paper with all new text marked in blue. The line numbers given in comments below correspond to this PDF.

In the resubmission we have included a file Fontaine_et_al_diff,pdf showing the changed text and associated deletions and, as it may be easier to read, a file Fontaine_et_al_diff_addonly.pdf that only marks the new text and not the deletions.

**Reviewer 1.**

*This manuscript introduces Dynamic Landscape Analysis, a framework to build predictive models from single-cell data. This work aligns with current approaches aimed at constructing "landscape models," and demonstrates, through a specific example, how such models can be built and interpreted, leading to new predictions.*

*In my opinion, this manuscript represents an important step forward in both the theoretical and computational understanding of differentiation. The study is very thorough and rigorous, and I think PLOS Biology could be an excellent venue to advertise the ideas presented and showcase their concrete applications, with very clear predictions. It is among the most rigorous approaches currently available to think about these systems. Given the current "landscape" of the field, the general approach and the results presented, I am in favour of eventual publication.*

Thank you. We are grateful for the reviewer's appreciation.

*That said, in my opinion, the main issue with the manuscript is the writing. The current paper is not sufficiently targeted at a general biology audience, and I think that only readers with a rather advanced mathematical background will be able to fully understand it. I appreciate that the problem the authors are trying to solve is very difficult and requires a substantial amount of technical explanation to be fully rigorous. However, I am concerned that many readers will struggle to follow the logic and details of the paper, which is very dense and often highly jargonic. For example, I would expect interested readers to already be familiar with terms such as "bifurcations" or "normal forms," but more specialized nomenclature may confuse many readers. I am thinking in particular of terms such as "bifurcation set" or "fold curves," which are transparent only to those with a strong background in the underlying mathematics. The authors also introduce their own jargon, for instance the notion of a "head attractor" for binary flips. While people familiar with the field will likely understand these terms, I suspect they may confuse the majority of potential readers. This also tends to obscure very basic questions about the formalism, which would be helpful to address more explicitly. I also think that some very basic aspects of the formalism and of the modelling choices should be discussed earlier in the manuscript.*

We accept the reviewer's criticism and therefore have revised the text to make it more accessible for a general audience. Firstly, we surveyed the paper for all mathematical terms and other jargon. Where possible we replaced them (e.g. we replaced "heteroclinic" by "a direct transition pathway linking two saddle points") or provided brief explanatory commentary where they are used (e.g. replaced "Kullback–Leibler Divergence" with "Kullback–Leibler Divergence (a statistical measure of how well-separated two clusters are))".

A small number of terms that biologists might be unfamiliar with are so central to our discussion that we have kept them and treated this issue by including three explanatory boxes. These contain a compact discussion of their meaning in the developmental context including figures to aid understanding. Examples of such terms include landscape, attractor, saddle, bifurcation, fold and flip. As well as outlining the meaning of such terms, the boxes allow a more complete discussion of their relevance for cellular development. This brings together more coherently pieces of discussion that were scattered around the manuscript and therefore improves the readability but does not increase the length of the text by too much because the boxes replace text that was present in the body of the manuscript.

We need to keep these terms because a central point of our paper is that precise mathematical notions underlie the biological observations and motivate the methods we use to analyse the data. For example, we assert that there are powerful mathematical results that strongly suggest that the transition routes between cell states correspond to the 1-dimensional unstable manifolds that connect the attractors of the dynamical system defined by the relevant gene regulatory network. We could replace "unstable manifold" by "transition pathway" or some similar phrase throughout the manuscript, but this would cause confusion, as it would not be clear whether we were referring to the mathematical notion or the actual transition pathway observed in the data and it would remove the crucial idea that the mathematical notions underlie the way we analyse the biological observations.

The three boxes address aspects of the paper that the reviewer raises. The first gives an overview of the dynamical systems approach to development and introduces the most basic ideas. The second introduces and explains the bifurcations that we find in the data, particularly those associated with branching in the developmental pathways. The third explains the notion of a bifurcation set to help the reader with the part of the paper dealing with model construction. In the box title we have included a list of the terms explained so that readers an quickly find a term’s meaning/interpretation when reading the paper.

The section describing the model construction has been rewritten (lines 333-350) so that the part requiring a number of technical terms is covered in the SI rather than in the main paper.

*The entire landscape description is based on the idea that cells follow escape paths determined by the saddle's unstable manifold, and this property is then used to infer the relative locations of saddles and attractors. This process is not explained in sufficient detail. I am confident that the authors can look at the data and immediately identify where the saddles should be, but I suspect that many readers will not see how these choices are made, beyond a qualitative sense that "the figures look similar" (e.g. Fig. 3E vs Fig. 3F). I think the authors should devote more time to clearly explaining what happens at this stage, how specific landscapes are selected, and what criteria are used. For instance, when the authors write that there is "strong support for the binary flip architecture," it is not clear which specific aspects of the data provide this strong support (what would be the alternative, or what would constitute weaker evidence?). In previous papers, a distinction was made between flip and choice architectures; perhaps the authors could make this distinction more explicit here.*

We have addressed this point by a number of significant additions and modifications. The most significant is the discussion in lines 203-238 which gives more context and more details of how we extract transition pathways from the data and, where there is branching, how we distinguish between the alternative models for this (essentially the binary flip and the binary choice). The information in Box 2 also helps the reader's understanding of this. The criteria used for validation are clearer and are more fully described in this version.

We also make it clearer that our task in this section is not to determine the exact position of saddles, etc., as they will vary from cell to cell, but to identify the nature of the underlying bifurcation involved. To do this we must (i) identify the genes that vary significantly along the transition routes (ii) determine how their expression varies as a function of time, and crucially, (iii) validate the assertion that the temporal gene expression of calls along a potential transition pathway falls into one of two patterns, and (iv) apply criteria (explained in 228 -232) to distinguish between a binary flip and a binary choice.

Our approach in this section represents a significant methodological advance over our previously published work, which relied on lower-dimensional flow cytometry data and lacked the ability to directly observe the geometry of transitioning cell populations in the higher dimensional transcriptional space.

*More fundamentally, how do we know that the attractors identified are "real" attractors? I am struck by the fact that "head attractors" are often very close to saddles, which is necessary to generate the observed dynamics. However, if noise is added, can we fully exclude other possibilities, such as ghost fixed points or very flat valleys? Other groups have suggested that ghost fixed points may play a role in development, and it would be useful to discuss how such alternatives are ruled out or distinguished here.*

The questions raised here are treated in two significant passages in lines 155-165 and 601-629. The first of these was not previously sufficiently highlighted in the manuscript. It concerns the reproducibility of our data across multiple time points and experimental conditions, and the way we can use multiple observations on a putative attractor to determine whether it's really there. The latter discussion is about how this reproducibility is used, the need for perturbation experiments to resolve this question, and the use of the ideas in lines 239-245 on the temporal progression of cells transitioning along the unstable manifolds to distinguish between ghost states and real but shallow attractors. This approach also addresses the question about flat valleys. Finally, we point out that our fitting of the model to the data provides an independent check on the possibility that the attractor is a ghost, is leaking or is a deep attractor.

Briefly, comparing the different ACs across multiple conditions (times, Shh levels) directly in gene expression space, LDA projections and on the standard 2D plots of expression levels of pairs of key genes, indicated that they are reproducible across conditions except when they bifurcate. We can then verify that the sub-landscapes containing a source AC and the small number of potential downstream ACs, are stable across conditions and investigate the stability of the source AC under some conditions and the emptying of it under others. Occupation of an AC over multiple time points or a clear bifurcation event indicates that the attractor is not a ghost, but in some cases, more definitive proof requires perturbation experiments.

*While smooth trajectories within tubular neighbourhoods, such as those shown in Fig. 3H, are compatible with the data, there is an implicit assumption that should be made more explicit. We only observe snapshots of trajectories, so it is conceivable that cells could switch between the red and blue paths in Fig. 3F. There could also be shortcuts in higher dimensions, and our representation may be biased by the choice of linear discriminant representation. Until cells can be followed directly within the landscape, we cannot be fully confident that snapshots of cell distributions correspond to the actual trajectories depicted (with some noise).*

This needs a little unpacking. We are not assuming that cells cannot switch between transition paths, indeed we would expect this to happen in such a stochastic system. However, once the paths have diverged, we would expect this to be rare (as it would require a large stochastic fluctuation) and they would be greatly dominated by the cell transitions that are along one of the unstable manifold routes. This is the case in simulations where the level of the noise is reasonable (not too high). As the reviewer suggests, to understand how often this occurs in experiments would require following cells in the landscape using live cell imaging rather than snapshots. We have added a comment about this in lines 621-632.

Regarding shortcuts in higher dimensions, presumably this comment refers to the possibility that these would be missed because the genes that change along the shortcut are not represented in our gene expression space. We have added a comment about this in the discussion (lines 623-632). We feel that this is less likely, as we would pick up any of those extra genes that are differentially expressed between the ACs since, as mentioned in the manuscript, we test for them after identifying the AC clusters. Moreover, if the cells in this route were plentiful this would affect the proportions occupying each of the relevant cell states making it hard for the model to consistently fit the data.

In case there is a misunderstanding, we emphasise the point that the putative unstable manifolds are not inferred from the two-dimensional LDA visualisations but are assessed in the higher-dimensional gene expression space defined by the full set of selected genes. We have ensured this is highlighted in the revised version.

*The statement "However, the data indicated that the downstream states pMN and DP are part of a linear structure consisting of the four ACs MNDiff, pMN, DP and p3 with saddles between each of these (Fig. 5B)" raised a similar concern. I wondered whether such observations might partly reflect the representation choice (Fig. 5B being a theory-driven figure).*

This statement of linearity relies on the criteria given in 220-232. Using splines fitted to the transitioning cells, as in 203-216, we investigated the route of departure from the head AC. In contrast to cells exiting along a shared initial direction before diverging, we observed a coherent gene expression profile and departures in different directions from the AC, consistent with two distinct saddles either side of the AC. This has now been clarified in the manuscript.

Importantly, this section describing where we find a binary choice transition (310-322) has been clarified using the criteria developed earlier in the paper (using splines fitted to the transitioning cells (lines 203-216) to validate departures in different directions from the AC, consistent with two distinct saddles either side of the AC).

*For Fig. 5, I would suggest always placing the "head attractor" at the top of each subpanel, to remain consistent with the overall tree-like architecture.*

Agreed. Thank you.

*For Fig. 6, stitching landscapes together defines an overall 2D manifold. I was wondering whether the presence of the repeller is a consequence of this effective 2D representation. Would one necessarily obtain a repeller in very high-dimensional spaces?*

The repeller is a necessary consequence of the presence of a circular structure in a 2D landscape. This follows from the so-called Morse-Smale inequalities. In higher-dimensional state spaces, this implied fixed point would be an index-2 saddle, i.e. a saddle whose unstable manifold is 2-dimensional.

*Finally, to some extent, several points made in the Discussion could be introduced earlier to better motivate the landscape framework. For instance, statements such as "Cells can either remain in a strong attractor until a signal triggers a bifurcation, or linger near a shallow attractor for an extended period before escaping through stochastic fluctuations (Fig. 7B)" are fundamental to justifying the landscape approach itself. Similarly, "An important implication of the flip bifurcation is that a cell first leaves the head attractor before determining which downstream fate it adopts. In other words, in these transitions, a cell commits to differentiate before specifying which fate it will choose." This is a strong argument in favour of using binary flips (rather than binary choices) for the data presented. I suspect that this point constitutes part of the "strong evidence" for binary flips mentioned earlier in the text, and as such it would be helpful to introduce it earlier.*

Thank you for these suggestions. We have moved key conceptual points that are currently in the Discussion into the Introduction or Results.

**Reviewer 2.**

*I must congratulate the authors on this rigorous and robust study: almost every primary analysis and model testing in this manuscript (which are several) is supported by several secondary analyses and tested for robustness.*

Thank you. We are grateful for the reviewer's appreciation.

*I suggest that the authors split this into two studies (if the editor allows) , e.g. Part 1 and Part 2, for ease of readability (even in that case, I recommend simplifying figures). I personally would not mind this excellent study be published as two papers. Alternatively, various sections of the study should be moved to supplementary.*

While we understand the reasons for this suggestion, we would prefer that the study remains a single manuscript. Our view is that the strength of the study is the integrated analysis, from data to predictive model. Our view is that it would be difficult to communicate the key findings of the approach if we were to split it into two papers. We have extensively edited the text in an attempt to improve accessibility and flow. We hope this will help readers.

*Analysis in lines 113-onward: DId the authors ensure that this was not a simple result of subsampling within scRNAseq datasets. What were the count thresholds?*

This meaning of this comment is not immediately clear to us i.e. we are not clear about what the "this" in "this was not a simple result of subsampling" is referring to. We interpret it as questioning whether the set of about 100 genes identified by the process described is only a smallish number because of subsampling. This was addressed in the paragraph later in this section where we confirmed, after the clustering to find ACs and transitioning groups, that in this group we had captured the genes that are differentially expressed between each cluster and the others and, in addition, confirmed robustness of the group by substituting alternative correlated marker genes and verifying that cluster structures remained consistent. Moreover, after analysing transition pathways, we expanded the gene set to include genes showing significant variation in transitions, along with expert-curated transcription factors. None of these additions substantially affected subsequent analyses. When we start the process, we do not assume any cell states and therefore intended this gene selection process as a bootstrapping, but found that for our system, the first step hardly needed any further refinement.

*Many figures have too many panels, often distracting to me (e.g. bar plots in Fig. 8). Oftentimes, the font size is too small, limiting readability, on top of too many panels.*

We have gone through the figures increasing font size where possible. In some the number of subfigures has been reduced because they have been incorporated into boxes. We very seriously considered reducing the bar plots in Fig. 8, but decided against it because we believe that the level of accuracy with which the simulations match the data will be of interest to most readers, and we felt it should be readily available. We could not come up with any better method of doing this.

*Some terms are used without appropriate explanation: e.g. what contour threshold should be used to define leaking vs emptied. Greater formalism would be beneficial.*

We have been more explicit about this throughout the paper. We define an AC as leaking when its cell density decreases over time, indicating sustained net flux out of its basin. An AC is considered emptied when its density falls below 5% of its original population, indicating effective loss of occupancy over time. To distinguish shallow from bifurcated ACs, we classify an AC as shallow if, despite leaking, it still retains at least 10% of its original population after 48 h and this time scale depends on the biological system under study. By contrast, a bifurcated AC is one that does not meet this retention criterion and instead shows substantial depletion as cells transition into downstream states. In addition, we use the technique illustrated in Fig. 3K to study the temporal progression of cells transitioning along the unstable manifolds of this landscape. Among other things, this distinguishes a bifurcated AC from one that is leaking as when leaking the distribution is in equilibrium while when bifurcated there is a pulse of cells moving down the unstable manifold.

*Lineage tracing as a more concrete experimental way to test their model on transitions and paths should be covered in discussion. Their in vitro model is particularly amenable to such approaches. Note that I do not expect the authors to perform those experiments in this study.*

We agree with the referee. Combining DLA with clonal/lineage analysis is likely to provide additional insight into transition paths and landscape geometry. As the referee notes, this is beyond the scope of the current study but it is on our agenda.

*Lines 150-162. Instead of quoting entire panels in one sentence (1G-L), I recommend the authors mention each panel clearly next to the respective discussion.*

We have done this.

*It is unclear what the "FACS" data is: how many genes/markers were sorted for? Also, is it generated for this study or used from existing data (Delas et al?)? Clearly stating what is generated in-house vs re-used is important, but currently lacking. My view doesn't change even if all analysis is done on published data, but clarify*.

We refer to this now as flow cytometry data as the use of FACS implies that we used cell sorting which is not the case. The flow cytometry data were generated for this work. The cells were analysed, not sorted. New experiments were performed due to the lower resolution in the Delas et al data (ref [36] in manuscript), where the cells underwent additional treatments for genomic assays and sorting. We have clarified this in the Methods section.

The section "The effect of changing Shh levels on the landscape", where the flow cytometry data appears for the first time, details the makers used (Sox2, Pax6, Olig2, Nkx6.1, and Nkx2.2).

*The study uses too many acronyms which further affects readability*.

We removed the acronyms that were unnecessary and kept them to a minimum in the main text. In particular, we use only DLA (dynamic landscape analysis), ACs (attractor clusters), LDA (linear discriminant analysis), and PPDs (posterior parameter distributions). We explain LDA in the SI but do not expect it to cause confusion, as it is almost as widely used as PCA (Principal Component Analysis). Standard acronyms such as scRNA-seq for transcriptomic data are well established terminologies and should not confuse the readers. The remaining acronyms refer to cell states, namely MN (motor neurons), pMN (motor neuron progenitors), NMP (neuromesodermal progenitors), and FP (floor plate).

*Line 146: what is the typical absolute values for gene-gene correlations within and between ACs that the authors observe?*

The correlations we find span two orders of magnitude in amplitude, from effectively 0.01 (not significantly different from zero) to highly significant at around 0.5. In the plots in Appendix A, they are coloured by level to aid identification and marked by significance level. We calculate correlations only within ACs, and the correlations we study are those found in fluctuations from the mean within the ACs. In a stochastic differential equation, the correlation matrix at an AC can be calculated directly in terms of the Jacobian at the corresponding attractor and the noise structure, showing how aspects of the regulatory network encoded in the Jacobian will be reflected in the correlation matrix.

9.6.26
