# Supplementary material for: Dynamic Landscape Analysis of cell fate decisions provides predictive models of neural development from single-cell data
Source: PLoS Biol. 2026 Aug 26;24(8):e3003953. doi: 10.1371/journal.pbio.3003953 (PMC13588503; doi:10.1371/journal.pbio.3003953)
Supplement: Peer review history

# Editor report: Decision Letter 0

**DOI:** 10.1371/journal.pbio.3003953.r001

**Authors:** Ines Alvarez-Garcia

16 Dec 2025

Dear James,

Thank you for submitting your manuscript entitled "Dynamic Landscape Analysis of Cell Fate Decisions: Predictive Models of Neural Development From Single-Cell Data" for consideration as a Research Article by PLOS Biology.

Your manuscript has now been evaluated by the PLOS Biology editorial staff as well as by an academic editor with relevant expertise and I am writing to let you know that we would like to send your submission out for external peer review.

However, before we can send your manuscript to reviewers, we need you to complete your submission by providing the metadata that is required for full assessment. To this end, please login to Editorial Manager where you will find the paper in the 'Submissions Needing Revisions' folder on your homepage. Please click 'Revise Submission' from the Action Links and complete all additional questions in the submission questionnaire.

Once your full submission is complete, your paper will undergo a series of checks in preparation for peer review. After your manuscript has passed the checks it will be sent out for review. To provide the metadata for your submission, please Login to Editorial Manager (https://www.editorialmanager.com/pbiology) within two working days, i.e. by Dec 18 2025 11:59PM.

If your manuscript has been previously peer-reviewed at another journal, PLOS Biology is willing to work with those reviews in order to avoid re-starting the process. Submission of the previous reviews is entirely optional and our ability to use them effectively will depend on the willingness of the previous journal to confirm the content of the reports and share the reviewer identities. Please note that we reserve the right to invite additional reviewers if we consider that additional/independent reviewers are needed, although we aim to avoid this as far as possible. In our experience, working with previous reviews does save time.

If you would like us to consider previous reviewer reports, please edit your cover letter to let us know and include the name of the journal where the work was previously considered and the manuscript ID it was given. In addition, please upload a response to the reviews as a 'Prior Peer Review' file type, which should include the reports in full and a point-by-point reply detailing how you have or plan to address the reviewers' concerns.

During the process of completing your manuscript submission, you will be invited to opt-in to posting your pre-review manuscript as a bioRxiv preprint. Visit http://journals.plos.org/plosbiology/s/preprints for full details. If you consent to posting your current manuscript as a preprint, please upload a single Preprint PDF.

Feel free to email us at plosbiology@plos.org if you have any queries relating to your submission.

Kind regards,

Ines

--

Ines Alvarez-Garcia, PhD

Senior Editor

PLOS Biology

ialvarez-garcia@plos.org

# Peer review: Decision Letter 1

**DOI:** 10.1371/journal.pbio.3003953.r002

**Authors:** Ines Alvarez-Garcia

13 Mar 2026

Dear James,

Thank you for your patience while your manuscript entitled "Dynamic Landscape Analysis of Cell Fate Decisions: Predictive Models of Neural Development From Single-Cell Data" was peer-reviewed at PLOS Biology. Please also accept again my sincere apologies for the delay in sending you the decision - as mentioned, we were unlucky with the reviewers and two of the ones we recruited didn't submit the report, forcing us to look for an additional one. The manuscript has now been evaluated by the PLOS Biology editors, an Academic Editor with relevant expertise, and by two independent reviewers.

The reviews are attached below. As you will see, the reviewers find the manuscript potentially interesting, but they also think that the writing is not accessible for our broad audience and should be significantly improved before we can consider the manuscript further for publication. Both reviewers provide detailed feedback on how to improve the writing, including explaining better the landscape description, how it is known that the attractors identified are ‘real’ attractors, and also note that the snapshot of cell distributions might not correspond to the actual trajectories shown. In addition, the reviewers highlight several statements that are confusing, mention that excessive jargon is used and think that both the Introduction and Discussion should be restructured to improve the flow of the manuscript.

In light of the reviews, we would like to invite you to revise the work to thoroughly address the reviewers' reports. Given the extent of revision needed, we cannot make a decision about publication until we have seen the revised manuscript and your response to the reviewers' comments. Your revised manuscript is likely to be sent for further evaluation by all or a subset of the reviewers.

In addition to these revisions, you will need to complete some formatting changes, which you will receive in a follow up email. A member of our team will be in touch with a set of requests shortly.

We expect to receive your revised manuscript within 3 months. Please email us (plosbiology@plos.org) if you have any questions or concerns, or would like to request an extension.

At this stage, your manuscript remains formally under active consideration at our journal; please notify us by email if you do not intend to submit a revision so that we may withdraw it.

**IMPORTANT - SUBMITTING YOUR REVISION**

Your revisions should address the specific points made by each reviewer. Please submit the following files along with your revised manuscript:

1. A 'Response to Reviewers' file - this should detail your responses to the editorial requests, present a point-by-point response to all of the reviewers' comments, and indicate the changes made to the manuscript.

*NOTE: In your point-by-point response to the reviewers, please provide the full context of each review. Do not selectively quote paragraphs or sentences to reply to. The entire set of reviewer comments should be present in full and each specific point should be responded to individually, point by point.

You should also cite any additional relevant literature that has been published since the original submission and mention any additional citations in your response.

2. In addition to a clean copy of the manuscript, please also upload a 'track-changes' version of your manuscript that specifies the edits made. This should be uploaded as a "Revised Article with Changes Highlighted" file type.

*Re-submission Checklist*

When you are ready to resubmit your revised manuscript, please refer to this re-submission checklist: https://plos.io/Biology_Checklist

To submit a revised version of your manuscript, please go to https://www.editorialmanager.com/pbiology/ and log in as an Author. Click the link labelled 'Submissions Needing Revision' where you will find your submission record.

Please make sure to read the following important policies and guidelines while preparing your revision:

*Published Peer Review*

Please note while forming your response, if your article is accepted, you may have the opportunity to make the peer review history publicly available. The record will include editor decision letters (with reviews) and your responses to reviewer comments. If eligible, we will contact you to opt in or out. Please see here for more details:

https://blogs.plos.org/plos/2019/05/plos-journals-now-open-for-published-peer-review/

*PLOS Data Policy*

Please note that as a condition of publication PLOS' data policy (http://journals.plos.org/plosbiology/s/data-availability) requires that you make available all data used to draw the conclusions arrived at in your manuscript. If you have not already done so, you must include any data used in your manuscript either in appropriate repositories, within the body of the manuscript, or as supporting information (N.B. this includes any numerical values that were used to generate graphs, histograms etc.). For an example see here: http://www.plosbiology.org/article/info%3Adoi%2F10.1371%2Fjournal.pbio.1001908#s5

*Blot and Gel Data Policy*

We require the original, uncropped and minimally adjusted images supporting all blot and gel results reported in an article's figures or Supporting Information files. We will require these files before a manuscript can be accepted so please prepare them now, if you have not already uploaded them. Please carefully read our guidelines for how to prepare and upload this data: https://journals.plos.org/plosbiology/s/figures#loc-blot-and-gel-reporting-requirements

*Protocols deposition*

To enhance the reproducibility of your results, we recommend that if applicable you deposit your laboratory protocols in protocols.io, where a protocol can be assigned its own identifier (DOI) such that it can be cited independently in the future. Additionally, PLOS ONE offers an option for publishing peer-reviewed Lab Protocol articles, which describe protocols hosted on protocols.io. Read more information on sharing protocols at https://plos.org/protocols?utm_medium=editorial-email&utm_source=authorletters&utm_campaign=protocols

Thank you again for your submission to our journal. We hope that our editorial process has been constructive thus far, and we welcome your feedback at any time. Please don't hesitate to contact us if you have any questions or comments.

Sincerely,

Ines

--

Ines Alvarez-Garcia, PhD

Senior Editor

PLOS Biology

ialvarez-garcia@plos.org

------------------------------------

Reviewers' comments

Rev. 1:

This manuscript introduces Dynamic Landscape Analysis, a framework to build predictive models from single-cell data. This work aligns with current approaches aimed at constructing "landscape models," and demonstrates, through a specific example, how such models can be built and interpreted, leading to new predictions.

In my opinion, this manuscript represents an important step forward in both the theoretical and computational understanding of differentiation. The study is very thorough and rigorous, and I think PLOS Biology could be an excellent venue to advertise the ideas presented and showcase their concrete applications, with very clear predictions. It is among the most rigorous approaches currently available to think about these systems. Given the current "landscape" of the field, the general approach and the results presented, I am in favour of eventual publication.

That said, in my opinion, the main issue with the manuscript is the writing. The current paper is not sufficiently targeted at a general biology audience, and I think that only readers with a rather advanced mathematical background will be able to fully understand it. I appreciate that the problem the authors are trying to solve is very difficult and requires a substantial amount of technical explanation to be fully rigorous. However, I am concerned that many readers will struggle to follow the logic and details of the paper, which is very dense and often highly jargonic. For example, I would expect interested readers to already be familiar with terms such as "bifurcations" or "normal forms," but more specialized nomenclature may confuse many readers. I am thinking in particular of terms such as "bifurcation set" or "fold curves," which are transparent only to those with a strong background in the underlying mathematics. The authors also introduce their own jargon, for instance the notion of a "head attractor" for binary flips. While people familiar with the field will likely understand these terms, I suspect they may confuse the majority of potential readers. This also tends to obscure very basic questions about the formalism, which would be helpful to address more explicitly. I also think that some very basic aspects of the formalism and of the modelling choices should be discussed earlier in the manuscript.

On a more scientific side:

* The entire landscape description is based on the idea that cells follow escape paths determined by the saddle's unstable manifold, and this property is then used to infer the relative locations of saddles and attractors. This process is not explained in sufficient detail. I am confident that the authors can look at the data and immediately identify where the saddles should be, but I suspect that many readers will not see how these choices are made, beyond a qualitative sense that "the figures look similar" (e.g. Fig. 3E vs Fig. 3F). I think the authors should devote more time to clearly explaining what happens at this stage, how specific landscapes are selected, and what criteria are used. For instance, when the authors write that there is "strong support for the binary flip architecture," it is not clear which specific aspects of the data provide this strong support (what would be the alternative, or what would constitute weaker evidence?). In previous papers, a distinction was made between flip and choice architectures; perhaps the authors could make this distinction more explicit here.

* More fundamentally, how do we know that the attractors identified are "real" attractors? I am struck by the fact that "head attractors" are often very close to saddles, which is necessary to generate the observed dynamics. However, if noise is added, can we fully exclude other possibilities, such as ghost fixed points or very flat valleys? Other groups have suggested that ghost fixed points may play a role in development, and it would be useful to discuss how such alternatives are ruled out or distinguished here.

* While smooth trajectories within tubular neighbourhoods, such as those shown in Fig. 3H, are compatible with the data, there is an implicit assumption that should be made more explicit. We only observe snapshots of trajectories, so it is conceivable that cells could switch between the red and blue paths in Fig. 3F. There could also be shortcuts in higher dimensions, and our representation may be biased by the choice of linear discriminant representation. Until cells can be followed directly within the landscape, we cannot be fully confident that snapshots of cell distributions correspond to the actual trajectories depicted (with some noise).

* The statement "However, the data indicated that the downstream states pMN and DP are part of a linear structure consisting of the four ACs MNDiff, pMN, DP and p3 with saddles between each of these (Fig. 5B)" raised a similar concern. I wondered whether such observations might partly reflect the representation choice (Fig. 5B being a theory-driven figure).

* For Fig. 5, I would suggest always placing the "head attractor" at the top of each subpanel, to remain consistent with the overall tree-like architecture.

* For Fig. 6, stitching landscapes together defines an overall 2D manifold. I was wondering whether the presence of the repeller is a consequence of this effective 2D representation. Would one necessarily obtain a repeller in very high-dimensional spaces?

* Finally, to some extent, several points made in the Discussion could be introduced earlier to better motivate the landscape framework. For instance, statements such as "Cells can either remain in a strong attractor until a signal triggers a bifurcation, or linger near a shallow attractor for an extended period before escaping through stochastic fluctuations (Fig. 7B)" are fundamental to justifying the landscape approach itself. Similarly, "An important implication of the flip bifurcation is that a cell first leaves the head attractor before determining which downstream fate it adopts. In other words, in these transitions, a cell commits to differentiate before specifying which fate it will choose." This is a strong argument in favour of using binary flips (rather than binary choices) for the data presented. I suspect that this point constitutes part of the "strong evidence" for binary flips mentioned earlier in the text, and as such it would be helpful to introduce it earlier.

Rev. 2: Yogesh Goyal - note that this reviewer has signed the review

This study uses (and develops new) dynamical systems modeling on scRNA-seq datasets from vertebrate neural tube development. They propose to use this framework, which they call dynamic landscape analysis, to infer topologies and transition paths within this developmental system, primarily in mESCs but eventually test their landscape model with realistic parameters on human data. They reach several quantitative conclusions, including (unexpectedly) multiple paths to convergent fates as well as experimentally demonstrate the predictive power of their model.

Overall, this is a tour de force manuscript on an important problem in stem cell and developmental biology, especially given the surge in single-cell RNAseq dataset production. The field needs formal frameworks, which this study successfully addresses. The conclusions are generally supported by the data presented and should be broadly useful to the community within development but also other fields, such as cancer biology. One overall comment, which shows up below in various forms, is that the manuscript and figures are quite dense (it would not be an overstatement to say that reviewing this study took me longer than any other studies I have reviewed before). Specific comments (in no particular order, including some positive and others minor) for the authors:

1. I must congratulate the authors on this rigorous and robust study: almost every primary analysis and model testing in this manuscript (which are several) is supported by several secondary analyses and tested for robustness.

2. I suggest that the authors split this into two studies (if the editor allows) , e.g. Part 1 and Part 2, for ease of readability (even in that case, I recommend simplifying figures). I personally would not mind this excellent study be published as two papers. Alternatively, various sections of the study should be moved to supplementary.

3. Analysis in lines 113-onward: DId the authors ensure that this was not a simple result of subsampling within scRNAseq datasets. What were the count thresholds?

4. Many figures have too many panels, often distracting to me (e.g. bar plots in Fig. 8). Oftentimes, the font size is too small, limiting readability, on top of too many panels.

5. Some terms are used without appropriate explanation: e.g. what contour threshold should be used to define leaking vs emptied. Greater formalism would be beneficial.

6. Lineage tracing as a more concrete experimental way to test their model on transitions and paths should be covered in discussion. Their in vitro model is particularly amenable to such approaches. Note that I do not expect the authors to perform those experiments in this study.

7. Lines 150-162. Instead of quoting entire panels in one sentence (1G-L), I recommend the authors mention each panel clearly next to the respective discussion.

8. It is unclear what the "FACS" data is: how many genes/markers were sorted for? Also, is it generated for this study or used from existing data (Delas et al?)? Clearly stating what is generated in-house vs re-used is important, but currently lacking. My view doesn't change even if all analysis is done on published data, but clarify.

9. The study uses too many acronyms which further affects readability.

10. Line 146: what is the typical absolute values for gene-gene correlations within and between ACs that the authors observe?

Yogesh Goyal

# Author comment: Author response to Decision Letter 2

**DOI:** 10.1371/journal.pbio.3003953.r003

15 Jun 2026

Attachment Submitted filename: Response to reviews.docx

# Peer review: Decision Letter 2

**DOI:** 10.1371/journal.pbio.3003953.r004

**Authors:** Ines Alvarez-Garcia

10 Jul 2026

Dear James,

Thank you for your patience while we considered your revised manuscript entitled "Dynamic Landscape Analysis of Cell Fate Decisions: Predictive Models of Neural Development From Single-Cell Data" for publication as a Research Article at PLOS Biology. This revised version of your manuscript has been evaluated by the PLOS Biology editors, the Academic Editor and the two original reviewers.

Based on the reviews, we are likely to accept this manuscript for publication, provided you satisfactorily address the data and other policy-related requests stated below my signature.

In addition, we would like you to consider a suggestion to improve the titled:

"Dynamic Landscape Analysis of cell fate decisions predicts models of neural development from single-cell data"

As you address these items, please take this last chance to review your reference list to ensure that it is complete and correct. If you have cited papers that have been retracted, please include the rationale for doing so in the manuscript text, or remove these references and replace them with relevant current references. Any changes to the reference list should be mentioned in the cover letter that accompanies your revised manuscript.

In addition to these revisions, you may need to complete some formatting changes, which you will receive in a follow up email. A member of our team will be in touch with a set of requests shortly. If you do not receive a separate email within a few days, please assume that checks have been completed, and no additional changes are required.

We expect to receive your revised manuscript within two weeks.

To submit your revision, please go to https://www.editorialmanager.com/pbiology/ and log in as an Author. Click the link labelled 'Submissions Needing Revision' to find your submission record. Your revised submission must include the following:

- a cover letter that should detail your responses to any editorial requests, if applicable, and whether changes have been made to the reference list

- a Response to Reviewers file that provides a detailed response to the reviewers' comments (if applicable, if not applicable please do not delete your existing 'Response to Reviewers' file.)

- a track-changes file indicating any changes that you have made to the manuscript.

NOTE: If Supporting Information files are included with your article, note that these are not copyedited and will be published as they are submitted. Please ensure that these files are legible and of high quality (at least 300 dpi) in an easily accessible file format. For this reason, please be aware that any references listed in an SI file will not be indexed. For more information, see our Supporting Information guidelines:

https://journals.plos.org/plosbiology/s/supporting-information

*Published Peer Review History*

Please note that you may have the opportunity to make the peer review history publicly available. The record will include editor decision letters (with reviews) and your responses to reviewer comments. If eligible, we will contact you to opt in or out. Please see here for more details:

https://plos.org/published-peer-review-history/

*Press*

Should you, your institution's press office or the journal office choose to press release your paper, please ensure you have opted out of Early Article Posting on the submission form. We ask that you notify us as soon as possible if you or your institution is planning to press release the article.

*Protocols deposition*

To enhance the reproducibility of your results, we recommend that if applicable you deposit your laboratory protocols in protocols.io, where a protocol can be assigned its own identifier (DOI) such that it can be cited independently in the future. Additionally, PLOS ONE offers an option for publishing peer-reviewed Lab Protocol articles, which describe protocols hosted on protocols.io. Read more information on sharing protocols at https://plos.org/protocols?utm_medium=editorial-email&utm_source=authorletters&utm_campaign=protocols

Please do not hesitate to contact me should you have any questions.

Sincerely,

Ines

--

Ines Alvarez-Garcia, PhD

Senior Editor

PLOS Biology

ialvarez-garcia@plos.org

------------------------------------------------------------------------

DATA POLICY:

You may be aware of the PLOS Data Policy, which requires that all data be made available without restriction: http://journals.plos.org/plosbiology/s/data-availability. For more information, please also see this editorial: http://dx.doi.org/10.1371/journal.pbio.1001797

Note that we do not require all raw data. Rather, we ask that all individual quantitative observations that underlie the data summarized in the figures and results of your paper be made available in one of the following forms:

1) Supplementary files (e.g., excel). Please ensure that all data files are uploaded as 'Supporting Information' and are invariably referred to (in the manuscript, figure legends, and the Description field when uploading your files) using the following format verbatim: S1 Data, S2 Data, etc. Multiple panels of a single or even several figures can be included as multiple sheets in one excel file that is saved using exactly the following convention: S1_Data.xlsx (using an underscore).

2) Deposition in a publicly available repository. Please also provide the accession code or a reviewer link so that we may view your data before publication.

Regardless of the method selected, please ensure that you provide the individual numerical values that underlie the summary data displayed in all relevant figure panels as they are essential for readers to assess your analysis and to reproduce it.

NOTE: the numerical data provided should include all replicates AND the way in which the plotted mean and errors were derived (it should not present only the mean/average values).

Please also ensure that figure legends in your manuscript include information on WHERE THE UNDERLYING DATA CAN BE FOUND, and ensure your supplemental data file/s has a legend.

Please ensure that your Data Statement in the submission system accurately describes where your data can be found.

------------------------------------------------------------------------

CODE POLICY

Per journal policy, if you have generated any custom code during the course of this investigation, please make it available without restrictions. Please ensure that the code is sufficiently well documented and reusable, and that your Data Statement in the Editorial Manager submission system accurately describes where your code can be found. More information on our Code Policy, what and how to share can be found here: https://journals.plos.org/plosbiology/s/code-availability

Please note that we cannot accept sole deposition of code in GitHub, as this could be changed after publication. However, you can archive this version of your publicly available GitHub code to Zenodo. Once you do this, it will generate a DOI number, which you will need to provide in the Data Accessibility Statement (you are welcome to also provide the GitHub access information). See the process for doing this here: https://docs.github.com/en/repositories/archiving-a-github-repository/referencing-and-citing-content

------------------------------------------------------------------------

Reviewers' comments

Rev. 1:

The authors have addressed all my comments. I would like to congratulate them for this wonderful study !

Rev. 2: Yogesh Goyal

The authors have addressed my comments and clarifications.

# Author comment: Author response to Decision Letter 3

**DOI:** 10.1371/journal.pbio.3003953.r005

24 Jul 2026

Attachment Submitted filename: Response_to_reviews_auresp_3.docx

# Editor report: Decision Letter 3

**DOI:** 10.1371/journal.pbio.3003953.r006

**Authors:** Ines Alvarez-Garcia

31 Jul 2026

Dear James,

Thank you for the submission of your revised Research Article entitled "Dynamic Landscape Analysis of cell fate decisions provides predictive models of neural development from single-cell data" for publication in PLOS Biology. On behalf of my colleagues and the Academic Editor, Marianne Bronner, I am delighted to let you know that we can in principle accept your manuscript for publication, provided you address any remaining formatting and reporting issues. These will be detailed in an email you should receive within 2-3 business days from our colleagues in the journal operations team; no action is required from you until then. Please note that we will not be able to formally accept your manuscript and schedule it for publication until you have completed any requested changes.

Please take a minute to log into Editorial Manager at http://www.editorialmanager.com/pbiology/, click the "Update My Information" link at the top of the page, and update your user information to ensure an efficient production process.

PRESS

We frequently collaborate with press offices. If your institution or institutions have a press office, please notify them about your upcoming paper at this point, to enable them to help maximise its impact. If the press office is planning to promote your findings, we would be grateful if they could coordinate with biologypress@plos.org. If you have previously opted in to the early version process, we ask that you notify us immediately of any press plans so that we may opt out on your behalf.

We also ask that you take this opportunity to read our Embargo Policy regarding the discussion, promotion and media coverage of work that is yet to be published by PLOS. As your manuscript is not yet published, it is bound by the conditions of our Embargo Policy. Please be aware that this policy is in place both to ensure that any press coverage of your article is fully substantiated and to provide a direct link between such coverage and the published work. For full details of our Embargo Policy, please visit http://www.plos.org/about/media-inquiries/embargo-policy/.

Many congratulations and thanks again for choosing PLOS Biology for publication and supporting Open Access publishing. We look forward to publishing your study.

Sincerely,

Ines

--

Ines Alvarez-Garcia, PhD

Senior Editor

PLOS Biology

ialvarez-garcia@plos.org
